# Salivary gland protective and antiinflammatory effects of genistein in Sjögren’s syndrome by inhibiting *Xist*/ACSL4-mediated ferroptosis following binding to estrogen receptor-alpha

**DOI:** 10.1186/s11658-024-00667-6

**Published:** 2024-12-02

**Authors:** Tianjiao Mao, Wei Wei, Bo Chen, Yixin Chen, Shuqi Liang, Guiping Chen, Zhuoyuan Liu, Xiaodan Wu, Lihong Wu, Xiaomeng Li, Nobumoto Watanabe, Kevin H. Mayo, Janak L. Pathak, Jiang Li

**Affiliations:** 1 School and Hospital of Stomatology, Guangdong Engineering Research Center of Oral Restoration and Reconstruction, Guangzhou Medical University, Guangzhou, 510140 China; 2 Department of Prosthodontics, School and Hospital of Stomatology, Jilin University, Changchun, 130012 China; 3 KingMed School of Laboratory Medicine, Guangzhou Medical University, Guangzhou, 510140 China; 4 Bioprobe Application Research Unit, RIKEN-Max Planck Joint Research Division, RIKEN Center for Sustainable Resource Science, Wako, Saitama 351-0198 Japan; 5 Graduate School of Medical & Dental Sciences, Tokyo Medical and Dental University, Tokyo, Japan; 6 Biochemistry, Molecular Biology, and Biophysics, Health Sciences Center, University of Minnesota, Minneapolis, MN 55455 USA

**Keywords:** Genistein, Sjögren’s syndrome, Salivary gland epithelial cells, Ferroptosis, *XIST*, ACSL4

## Abstract

**Background:**

Sjögren’s syndrome (SS) is an autoimmune disease with limited effective treatment options. This study aimed to explore the underlying mechanism by which genistein–estrogen receptor alpha (ERα) complex targets X-inactive specific transcript (*Xist*) then leads to the inhibition of ferroptosis by regulating acyl-CoA synthetase long-chain family member 4 (ACSL4) expression in salivary gland epithelial cells (SGECs) to attenuate SS.

**Methods:**

The effects of genistein treatment on the progression and underlying mechanism of SS were investigated using nondiabetic obese (NOD)/LtJ mice in vivo and Interferon-γ (IFNγ)-treated SGECs in vitro. Water intake and saliva flow rate were measured to evaluate the severity of xerostomia. Hematoxylin–eosin staining, real-time quantitative polymerase chain reaction, and enzyme-linked immunosorbent assay were conducted to examine the pathological lesions. Western blotting and immunohistochemistry analysis were used to evaluate the protein expression. RNA sequencing and RNA fluorescence in situ hybridization were employed to verify the relationship between *Xist* and ACSL4. Surface plasmon resonance, molecular docking, and molecular dynamics were used to investigate the binding between genistein and ERα. Furthermore, a chromatin immunoprecipitation assay was used to analyze ERα–*XIST* promoter interactions. The levels of malondialdehyde, glutathione, Fe^2+^, and mitochondrial changes were measured to evaluate ferroptosis of SGECs.

**Results:**

In NOD/LtJ mice, a ferroptosis phenotype was observed in salivary glands, characterized by downregulated *Xist* and upregulated X chromosome inactivation gene *Acsl4*. Genistein significantly alleviated SS symptoms, upregulated the *Xist* gene, and downregulated *Acsl4* expression. Genistein upregulated *Xist* expression in the salivary gland of NOD/LtJ mice via the ERα signaling pathway. It downregulated *Acsl4* and ferroptosis in the salivary glands of NOD/LtJ mice. IFNγ-treatment induced inflammation and ferroptosis in SGECs. Genistein binding to ERα upregulated *XIST*, and aquaporin 5 expression, downregulated ACSL4, and SS antigen B expression, and reversed ferroptosis in SGECs. Genistein mitigated inflammation and ferroptosis in SGECs by upregulated-*XIST*-mediated ACSL4 gene silencing.

**Conclusions:**

Genistein binding to ERα targets *Xist*, leading to inhibiting ferroptosis by regulating ACSL4 expression in SGECs. This finding provides evidence for genistein as a treatment for SS and identifies *Xist* as a novel drug target for SS drug development, offering great promise for improving SS outcomes.

**Graphical Abstract:**

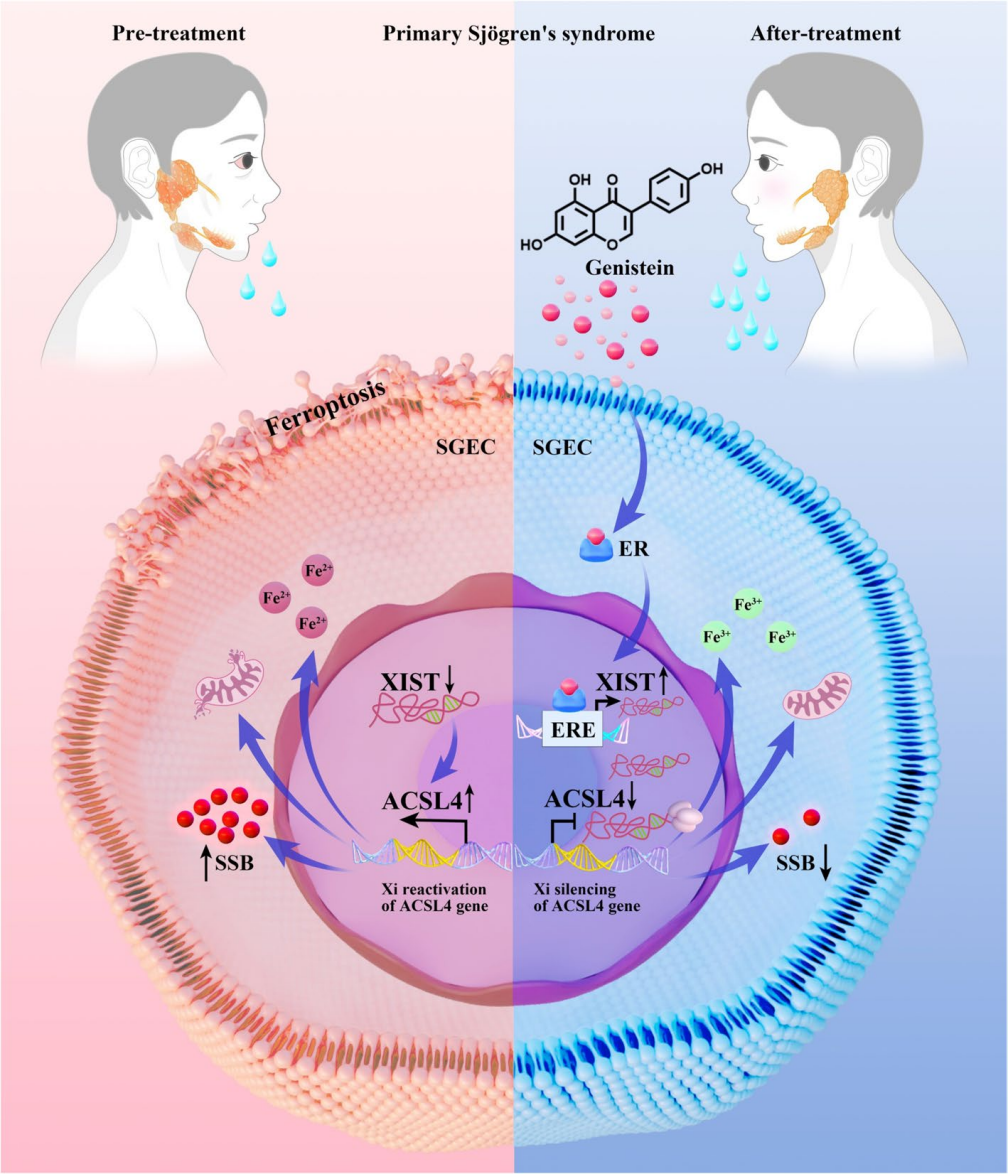

**Supplementary Information:**

The online version contains supplementary material available at 10.1186/s11658-024-00667-6.

## Background

Sjögren’s syndrome (SS) exhibits the most pronounced female preference among autoimmune diseases [1]. It is characterized by the production of autoantibodies (anti-SSA/Ro and anti-SSB/La) leading to inflammation, swelling, and damage to exocrine glands, primarily the salivary gland and lacrimal gland [2–4]. The phenotypic attributes of SS are heterogeneous; they primarily manifest as xerostomia and dry eye, which are often accompanied by systemic complications, including lung, renal, and nervous system involvement and lymphoma [5]. This clinical heterogeneity and unclear pathophysiological mechanism pose significant challenges to treatment and therapeutic advances. Although some advances in SS treatment have been made, patient outcomes remain suboptimal [6]. The morbidity accrual and toxicities of current regimens indicate that research for novel, effective, and targeted therapeutic approaches is urgently needed.

The health benefits associated with consuming plant-based foods have been attributed to flavonoids, a class of polyphenolic compounds commonly found in fruits, vegetables, tea, and red wine [7]. Several flavonoids including apigenin [8], quercetin [9], cardamonin [10], and epigallocatechin-3-gallate [11] have been extensively studied for their therapeutic potential in treating SS. Genistein is mainly found in soybeans and shows promise in alleviating symptoms of various autoimmune diseases [12]. It can inhibit angiogenesis in rheumatoid arthritis by targeting the interleukin (IL)-6/Janus kinase 2 (JAK2)/signal transducer and activator of transcription 3 (STAT3)/vascular endothelial growth factor (VEGF) signaling pathway [13] and also has the potential to treat primary biliary cholangitis [14] and multiple sclerosis [15]. However, its therapeutic effects in SS and the underlying mechanisms involved remain unclear.

A better understanding of the molecular features of SS that influence therapeutic response to therapy is essential. The X-inactive specific transcript (*Xist*), expressed on the inactivated X (Xi) chromosome, plays a key role in X chromosome inactivation (XCI) by forming a unique chromosomal conformation [16, 17], thereby maintaining the balance of sex-linked gene expression [18, 19]. Therefore, abnormal expression or mutation of the *Xist* gene may contribute to the onset of certain diseases, particularly female-biased diseases, such as autoimmune disorders [20]. However, the role of *Xist* in dysfunction of salivary gland epithelial cells (SGECs) and progression of SS remains unclear.

Ferroptosis is a nonapoptotic form of regulated cell death, characterized by excessive iron-dependent accumulation of lipid peroxidation products [21]. Emerging evidence indicates that ferroptosis is significantly involved in autoimmune diseases [22–24]. However, it remains unclear why ferroptosis occurs more prevalently in females and whether it is related to the female sex gene. The upregulation of long-chain acyl-CoA synthetase family member 4 (ACSL4), an enzyme involved in lipid metabolism and necessary for lipid peroxidation, promotes ferroptosis [25]. ACSL4 is fully inactivated on the XCI state [26]. The relationship between *XIST* and ferroptosis is not yet understood, and genistein-mediated regulation of ferroptosis in SGECs remains unclear.

Therefore, this study aims to investigate the therapeutic potential of genistein in SS by utilizing nonobese diabetic (NOD)/LtJ mice (an in vivo SS model) and interferon γ (IFNγ)-induced A253 cells (an in vitro SS model) [2]. Our findings reveal that genistein alleviates SS-related clinical and pathological features. Importantly, *Xist* was downregulated, and subsequently, ACSL4 was upregulated in SS models. Furthermore, we discovered that genistein binds to estrogen receptor alpha (ERα), which upregulates *Xist* expression. This leads to the downregulation of *Xist*-silenced-gene ACSL4 expression, thereby inhibiting ferroptosis in SGECs to attenuate SS.

## Materials and methods

### Animals and treatments

All animal studies were conducted under ethical policies and approved by the Animal Care and Use Committee at the Institute of Laboratory, Guangzhou Medical University (approval no. G2023-147, 2/3/2023). Female Institute of Cancer Research (ICR) mice (6–8 weeks old) and NOD/LtJ mice strain (N000235) (6–8 weeks old) were obtained from Gempharmatech (Nanjing, China). All mice were housed in a pathogen-free animal facility maintained under standard conditions and acclimatized to their environment for 7 days before starting experiments.

The mice were randomly divided into four groups, each containing six mice, including (I) control group, ICR mice given a basic diet and vehicle (0.5% sodium carboxymethyl cellulose (CMC-Na)); (II) model group, NOD/LtJ mice given a basic diet and vehicle; (III) genistein (50 mg/kg) group, NOD/LtJ mice given genistein (50 mg/kg) prepared with 0.5% CMC-Na solvent; (IV) hydroxychloroquine (HCQ, 50 mg/kg) group, NOD/LtJ mice given HCQ (50 mg/kg) prepared with 0.5% CMC-Na solvent. Respective formulations were administrated by gavage in all four murine groups for 8 weeks. Finally, followed by sacrifice, the peripheral blood from the ophthalmic vein was collected, and submandibular gland tissues were rapidly collected for analysis.

### Water intake and saliva flow rate assessment

Water intake was recorded weekly after drug administration; it was measured as water intake (mL) divided by body weight (g), while saliva flow rate was measured biweekly after an overnight fast as described previously [27]. Briefly, mouse was anesthetized with pilocarpine hydrochloride (0.1 mg/kg i.p.), then weight increase (mg) was calculated divided by body weight (g) during 10 min.

### Hematoxylin–eosin (H&E) staining and immunohistochemistry

The submandibular gland tissues embedded in paraffin were cut into 4-μm-thick slices and then stained with hematoxylin and eosin (H&E) to evaluate inflammatory cell infiltration and histopathological damage. For immunohistochemistry (IHC), the tissue sections were first blocked for 30 min, then incubated with primary antibodies, including anti-aquaporin 5 (AQP5, Abcam, ab305304) at a dilution of 1:100, and anti-ACSL4 (Santa Cruz, SC-365230) at a dilution of 1:50 at 4 ℃ for 16 h. The following day, slices were washed with PBS, followed by addition of the secondary antibody for 1 h at room temperature, followed by diaminobenzidine (DAB) solution incubation for 1 min and counterstaining with hematoxylin. Quantification of representative images at 100× magnification was carried out by an independent observer, Additionally, automated quantification was conducted using Image-Pro Plus software (version 6.0).

### Enzyme-linked immunosorbent assay

The serum levels of IFNγ, anti-SSA/Ro, and anti-SSB/La autoantibodies were detected using enzyme-linked immunosorbent assay kits (Mlbio, Shanghai, China) following the manufacturer’s instructions.

### Real-time quantitative polymerase chain reaction (RT-qPCR)

Total RNA was extracted using Trizol reagent, and complementary DNA (cDNA) was synthesized using the Evo M-MLV reverse-transcribed kit (Accurate Biology, Hunan, China) following the manufacturer’s protocol. The cDNA was subjected to RT-qPCR analysis using SYBR Green Pro Taq HS mix (Accurate Biology, Hunan, China). β-Actin was used as a standard control to analyze the relative expression mRNA levels according to the 2^(−ΔΔCt)^ method. All primer sequences are listed in Supplementary Table S1.

### RNA sequencing data analysis

RNA sequencing was performed at Biowefind (Wuhan, China). Raw data underwent processing with the robust multiarray mean algorithm (RMA) within the “Affy” package. Differentially expressed genes (DEGs) were identified using linear models from the “LIMMA” package in R language. Gene function classification and evaluation of biological functions were carried out using the Bioinformatics online tool. Heatmap, Kyoto Encyclopedia of Genes and Genomes (KEGG) enrichment, and Gene Ontology (GO) were performed to analyze the role of DEGs. A significance level of *p* < 0.05 was applied. Receiver operating characteristic (ROC) analysis was conducted using the “pROC” package in R to predict the diagnostic validity of biomarkers.

### RNA fluorescence in situ hybridization (FISH) assay

For RNA FISH, we used the *Xist*/*XIST* and *Acsl4*/*ACSL4* probes designed by GenePharma Co., Ltd. (Shanghai, China), with sequences listed in Supplementary Table S2. *Xist* and *Acsl4* RNA FISH in submandibular gland tissue or *XIST* and *ACSL4* RNA FISH in SGECs was performed using RNA FISH SA-Biotin kits (GenePharma) according to the manufacturer’s recommendations. Finally, slides were visualized using a fluorescence microscope (Olympus BX43, Olympus, Tokyo, Japan).

### Flow cytometry

Cells were incubated with fluorescent-conjugated antibodies for 15 min on ice to stain surface markers, then washed with cell staining buffer (BioLegend, San Diego, USA). Subsequently, Cells were fixed and permeabilized using a transcription-factor staining buffer, followed by a 30 min of incubation at room temperature with fluorescent-conjugated antibodies to stain intracellular antigens. The antibodies used for flow cytometry were APC anti-mouse FOXP3 (#32,007), FITC anti-mouse CD4 antibody (#100,411), and PE anti-mouse IL-17A (#116,107). All antibodies were purchased from BioLegend.

### Surface plasmon resonance analysis (SPR)

A Biacore 8K system (Cytiva, Marlborough, MA, USA) was used to analyze the direct interaction between ERα and genistein. ERα recombinant protein was immobilized on Series S Sensor Chip CM 5 (GE Healthcare Life, Chicago, USA) according to the manufacturer’s instruction. After that, different concentrations of genistein (12.5–200 μmol/L) were diluted in a running buffer and injected into the system as the analyte. The parameters for SPR were as follows: flow rate, 30 µL/min; association time, 60 s; dissociation time 90 s; temperature, 25 °C. Finally, the interaction parameters were obtained using Biacore evaluation software (version 2.0).

### Molecular docking and molecular dynamics (MD) simulations

We used AutoDock Vina for molecular docking. Initially, the ERα was prepared by deleting water molecules and bound ligands using the PyMol software. Subsequently, hydrogens were added, and energy minimization was performed using AutoDock. Finally, flexible ligands docking into the rigid binding site was conducted within the “Grid” module, and the Grid scores were used to estimate and rank ligand binding energies [28]. The optimal conformation with the lowest binding energy was selected as the initial conformation for MD simulations using Gromacs 2022.3 software as before [29]. The root-mean-square variance (RMSD), root-mean-square fluctuation, and protein rotation radius of each amino acid trajectory were calculated.

### Cell culture

The human SGEC-line A253 (ATCC Number: HTB-41) was purchased from Zhejiang Meisen Cell Biotechnology Co., Ltd. (ATCC no. HTB-41) (Zhejiang, China). Cells were cultured in RPMI-1640 (Hyclone, USA) medium supplemented with 10% fetal bovine serum (FBS, Gibco, USA) in a humidified atmosphere with 5% CO_2_ at 37 °C. The cells were passaged every three days.

### Cell viability assay

The assessment of cell viability in SGECs was conducted using Cell Counting Kit-8 (CCK-8) assays following the manufacturer’s protocols (MCE, Shanghai, China). These were performed at different concentrations of genistein (ranging from 0.001 µM to 100 µM), at specific time points of 24 h, 48 h, and 72 h post genistein intervention.

### Chromatin immunoprecipitation (ChIP) assay

The SGECs ChIP assay was performed according to the manufacturer’s protocol (BersinBio, Guangzhou, China). Sonicated lysates were incubated with 4 µg ERα (ab32063, Abcam) and mixed overnight at 4 °C. The next day, antibody-bound chromatin was incubated with Protein-G, washed with radioimmunoprecipitation assay (RIPA) buffer, and eluted in the elution buffer. The eluted sample was incubated at 65 °C overnight to reverse the crosslinking, and DNA was extracted using a TIANquick Midi purification kit (TIANGEN, DP204, Beijing, China) and subjected to PCR analysis. The primers ERE1 and ERE2 (Supplementary Table S3) were designed to amplify the *XIST* promoter region that contains ERα binding sites from the Jaspar database. After amplification, PCR products were resolved on a 1.5% agarose gel.

### Western blotting

Cells were lysed using RIPA buffer (Thermo Fisher Scientific, Waltham, MA, USA) containing protease inhibitors and phosphatase inhibitors (Beyotime, Nanjing, China), and protein concentrations were measured using a BCA protein assay kit (Thermo Fisher Scientific, Waltham, MA, USA). Total protein extracts (30 µg) were separated by sodium dodecyl sulfate (SDS)-polyacrylamide gel electrophoresis (PAGE) (Epizyme, Shanghai, China) using polyacrylamide gels and transferred onto polyvinylidene fluoride (PVDF) membranes (Millipore, Burlington, MA, USA). After being blocked with 5% skim milk for 1 h at room temperature, PVDF membranes were incubated with primary antibody overnight at 4℃. Then, they were incubated with secondary antibody for 1 h at room temperature. Finally, the protein was visualized by enhanced chemiluminescence reagents (Elabscience Biotechnology, Wuhan, China). Antibodies used in western blotting included: β-actin (ACTB, 1:10,000, Proteintech, 66,009–1-Ig), AQP5 (1:500, Santa Cruz, sc-514022), SSB (1:1000, Proteintech, 11,720–1-AP), and HRP-goat anti-rabbit/mouse secondary antibody (1:5000, Proteintech, RGAR001, RGAM001).

### Short interfering RNA (siRNA), short hairpin RNA (shRNA), and overexpression RNA (OE RNA) transfection

SiRNA, shRNA, OE RNA, and negative controls were designed and synthesized by GenePharma Co., Ltd. (Shanghai, China). To study the effect of *XIST* knockdown on ACSL4 and SS-like symptoms, 20 nM of siRNA targeting *XIST* was transfected into SGECs using EZ Trans (Life-ilab, Shanghai, China). For validation of ACSL4 inactivation by *XIST*-mediated gene silencing, 20 µg sh-split ends (SPEN) plasmid was transfected into SGECs. To validate the effect of ACSL4 in SS, sh-ACSL4 or OE-ACSL4 plasmid was transfected into SGECs. The method for using siRNA, sh-RNA, or OE-RNA transfection was done as specified by the manufacturer. Transfected cells were used for the experiments. The sequences of siRNA and plasmids used here are listed in Supplementary Table S4.

#### Measurement of malondialdehyde (MDA) and glutathione (GSH)

At the end of the experiments, SGEC lysates were collected. In these lysates, MDA and GSH were assessed by using the MDA (S0131, Beyotime) and GSH detection kits (S0103, Beyotime) according to the manufacturer’s instructions.

#### Transmission electron microscopy (TEM) assay

SG and SGEC samples were fixed in 2.5% glutaraldehyde and sent to Servicebio (Wuhan, China) for sample preparation and image acquisition.

#### Detection of intracellular Fe^2+^ levels in SGECs

Intracellular Fe^2+^ levels were detected using the FerroOrange probe (Dojindo, Tokyo, Japan) following the manufacturer’s instructions.

### Statistical analysis

All experiments were repeated at least three times. Data are shown as mean ± standard deviation (mean ± SD). Statistical analysis was performed using *t*-tests for comparison of two groups, and using one-way analysis of variance (ANOVA) followed by post hoc comparison for comparison between multiple groups. *p* < 0.05 was considered statistically significant. GraphPad Prism 8.0 (GraphPad Software, San Diego, CA, USA) was used for statistical analysis and figure preparation.

## Results

### Genistein alleviated Sjögren’s syndrome symptoms in NOD/LtJ mice

**Fig. 1:**
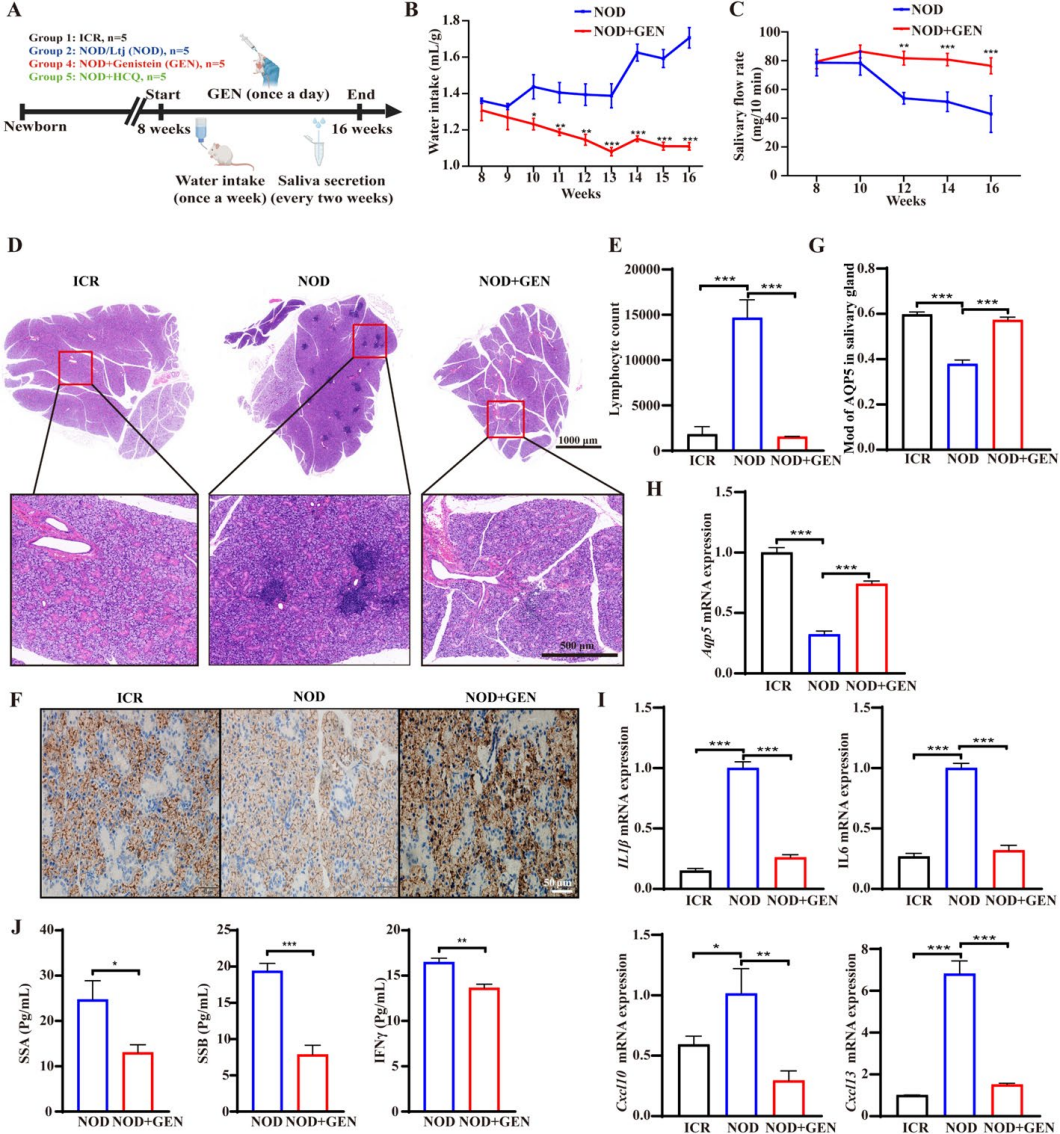
Genistein alleviated Sjögren’s syndrome symptoms in NOD/LtJ mice. **A** A schematic overview of animal experiments and treatment. **B** The assessment of water intake. **C** The assessment of salivary flow rate. **D**, **E** Hematoxylin–eosin (H&E) stained images and lymphocyte infiltration count in murine submandibular gland tissue. **F**, **G** Immunohistochemistry images and quantification of AQP5 of murine submandibular gland tissues. **H** mRNA expression of *Aqp5* in murine submandibular gland tissues. **I** mRNA expression of *Il1-β*, *Il6*, *Cxcl10*, and *Cxcl13* in murine submandibular gland tissues. **J** The levels of anti-SSA/SSB/IFN*γ* in NOD/LtJ mouse serum. Significant differences between groups are shown as: *p < 0.05, **p < 0.01, and ***p < 0.001; *n* = 5. NOD, nondiabetic obese (NOD)/LtJ; ICR, Institute of Cancer Research; GEN, genistein

First, we screened for interaction between ERα and 476 flavonoids. The molecular docking results showed that genistein exhibited the maximum affinity with ERα (Supplementary Table S5). We then assessed the effects of genistein in vivo (Fig. 1A). In untreated NOD/LtJ mice, water intake increased, whereas genistein significantly reduced water intake (Fig. 1B). Additionally, genistein increased the saliva flow rate in these NOD/LtJ mice (Fig. 1C), revealing that genistein reverses salivary gland dysfunction and improves thirst symptoms. Focal lymphocytic sialadenitis (FLS) is the primary histopathological feature of SS [30]. H&E staining showed that untreated NOD/LtJ mice exhibited the presence of acinar atrophy, various indications of chronic inflammation, and increased FLS located in perivascular or periductal site regions of the submandibular gland tissues. Genistein treatment ameliorated these histopathological features (Fig. 1D) and reduced the number of infiltrating lymphocytes in the submandibular glands of these mice (Fig. 1E).

Additionally, we investigated AQP5, a key protein involved in salivary gland water secretion. IHC and RT-qPCR analysis indicated that genistein significantly upregulated AQP5 expression in submandibular glands of NOD/LtJ mice, approaching the levels of AQP5 expression in ICR mice (Fig. 1F–H). RT-qPCR analysis demonstrated that genistein downregulates the expression of inflammation-related genes, including *Il1β*, *Il6*, *Cxcl10*, and *Cxcl13* (Fig. 1I). Furthermore, genistein reduced serum levels of anti-Ro/SSA and anti-La/SSB antibodies, as well as IFN*γ* in NOD/LtJ mice (Fig. 1J). Organ index analysis revealed that genistein decreased the submandibular gland index, spleen indices, and thymus index in NOD/LtJ mice (Fig. S1A). Furthermore, regulatory T (Treg) cells were upregulated and Th17 cells were downregulated in genistein-treated NOD/LtJ mice compared with untreated NOD/LtJ mice (Supplementary Fig. S1B,C). Interestingly, when the effect of genistein was compared with those of HCQ treatment, genistein showed superior efficacy in alleviating SS-related symptoms (Supplementary Fig. S2A–F). Moreover, genistein did not exhibit any adverse effects (Supplementary Fig S3). Our results indicate the therapeutic potential of using genistein to treat SS.

### Genistein binding to ERα upregulated *Xist* expression in submandibular glands of NOD/LtJ mice

**Fig. 2:**
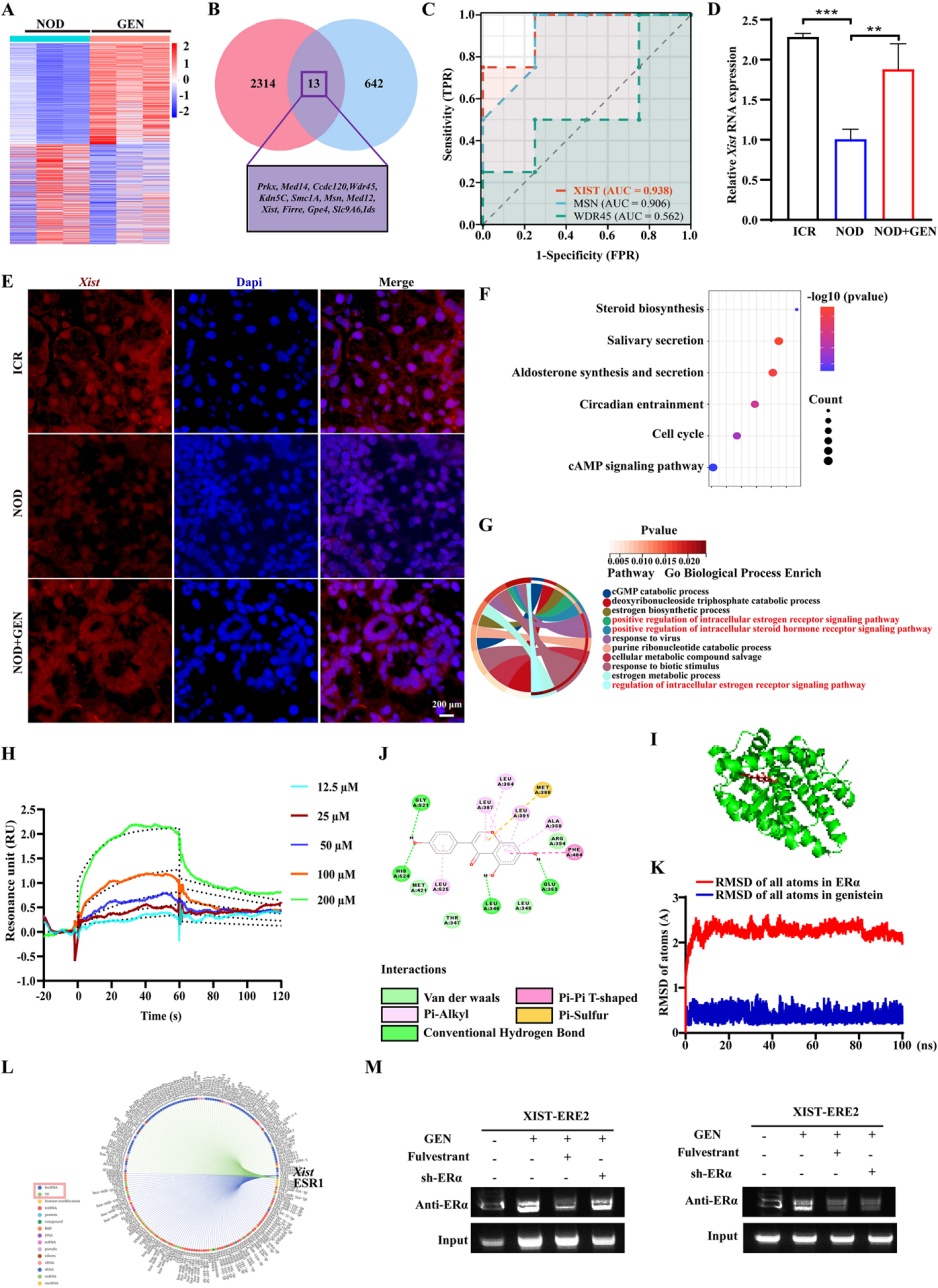
Genistein upregulated *XIST* expression via the ERα signaling pathway. **A** Heatmap of differentially expressed genes (DEGs) in the genistein treatment group, with DEGs screened based on *p* < 0.05 and |fold change|> 1. **B** Venn diagram of 13 overlapping DEGs among Chr X genes and upregulated genes in genistein-treated mice. **C** ROC curve analysis of 13 overlapping DEGs. **D** RNA expression levels of *Xist* in murine submandibular gland tissue. **E** *Xist* RNA fluorescence in situ hybridization (FISH) (red) in submandibular gland tissues of ICR, untreated NOD/LtJ, and genistein-treated NOD/LtJ mice. **F** The KEGG pathways attributed to DEGs. **G** The GO-enriched biological processes (BP) attributed to DEGs. **H** The SPR analysis of the direct interaction between genistein and ERα. **I** The ERα binding pocket of genistein. **J** Docking analysis between genistein and ERα protein (3ERD). **K** The RMSD values of genistein with ERα for 100 ns in MD simulation. **L** Prediction of ERα as an upstream regulator of *Xist*. Interactive networks of the top 100 genes interacting with *Xist* (www.rnainter.org/showMore/?raid=RP01930764). **M** ChIP assay of ERα in the conserved *Xist* promoter regions (CNS1 and CNS2) upon genistein and fulvestrant treatments or transfected with sh-ERα. Significant differences are indicated as: *p < 0.05, **p < 0.01, and ***p < 0.001; *n* = 5. ERα, estrogen receptor α; RMSD, root-mean-square deviation; MD, molecular dynamics

To uncover the genistein mechanism of action in SS, we conducted RNA-seq analysis. Genes were differentially expressed in the submandibular glands of untreated and genistein-treated NOD/LtJ mice (Fig. 2A). The resulting Venn diagram showed that there were 13 overlapping genes between ChrX genes and upregulated genes in the genistein group compared with the untreated group (Fig. 2B). The ROC curve suggested that *Xist* has the greatest sensitivity in SS (Fig. 2C). We then verified the expression of *Xist* in submandibular gland tissues by using RT-qPCR and RNA-FISH and found that genistein upregulates the *Xist* gene in SGECs in submandibular glands of NOD/LTJ mice (Fig. 2D, E). KEGG pathway analysis revealed that salivary secretion and steroid biosynthesis are top-enriched pathways (Fig. 2F). GO biological process analysis revealed that these genes were predominantly enriched in the estrogen biosynthesis process and positive regulation of the intracellular estrogen receptor (Fig. 2G). Together, this indicates a direct interaction between genistein and ERα protein.

Consistently, SPR analysis was used to ascertain the binding affinity of genistein with ERα; the result showed that genistein directly interacted with ERα in a concentration‐dependent manner (Fig. 2H). In addition, the docking simulation result showed that genistein interacted with the core cavity of the ERα active site (Fig. 2I), and genistein has a hydrophobic effect with ERα residues, including LEU346, GLU353, GLY521, and HIS524 (Fig. 2J). We further performed MD stimulation to investigate the dynamic movement and stability of genistein and ERα protein. The RMSD [31] values (~ 2.0 Å for ERα and ~ 0.2 Å for genistein) indicated that ERα binds stably to genistein in 0–100 ns (Fig. 2K). Furthermore, by using RNAInter (www.rnainter.org/showMore/?raid=RP01930764), we predicted that *XIST* transcription factors are involved, and found that ERα is one of the transcription factors that bind to *XIST* (Fig. 2L). Next, we analyzed the conserved sequences of *XIST* promoters from human genomes in the JASPAR database, and two conserved sequences were identified within the 2000 bp upstream from the transcription start site. In addition, we performed a CHIP assay to test whether ERα binds to *XIST* promoter regions. Notably, ERα showed significant upregulation in the two *XIST*-conserved promoter regions (ERE1 and ERE2) upon genistein treatment. With the knockdown of ERα or fulvestrant, we observed less ERα binding to ERE1 and ERE2 (Fig. 2M), indicating that genistein upregulates translation of *XIST* via the ERα pathway. Overall, we found that genistein alleviates SS symptoms by binding to ERα of SGECs, thereby upregulating *XIST* expression.

### Genistein alleviated ferroptosis via *Xist*-mediated *Acsl4* gene silencing

**Fig. 3:**
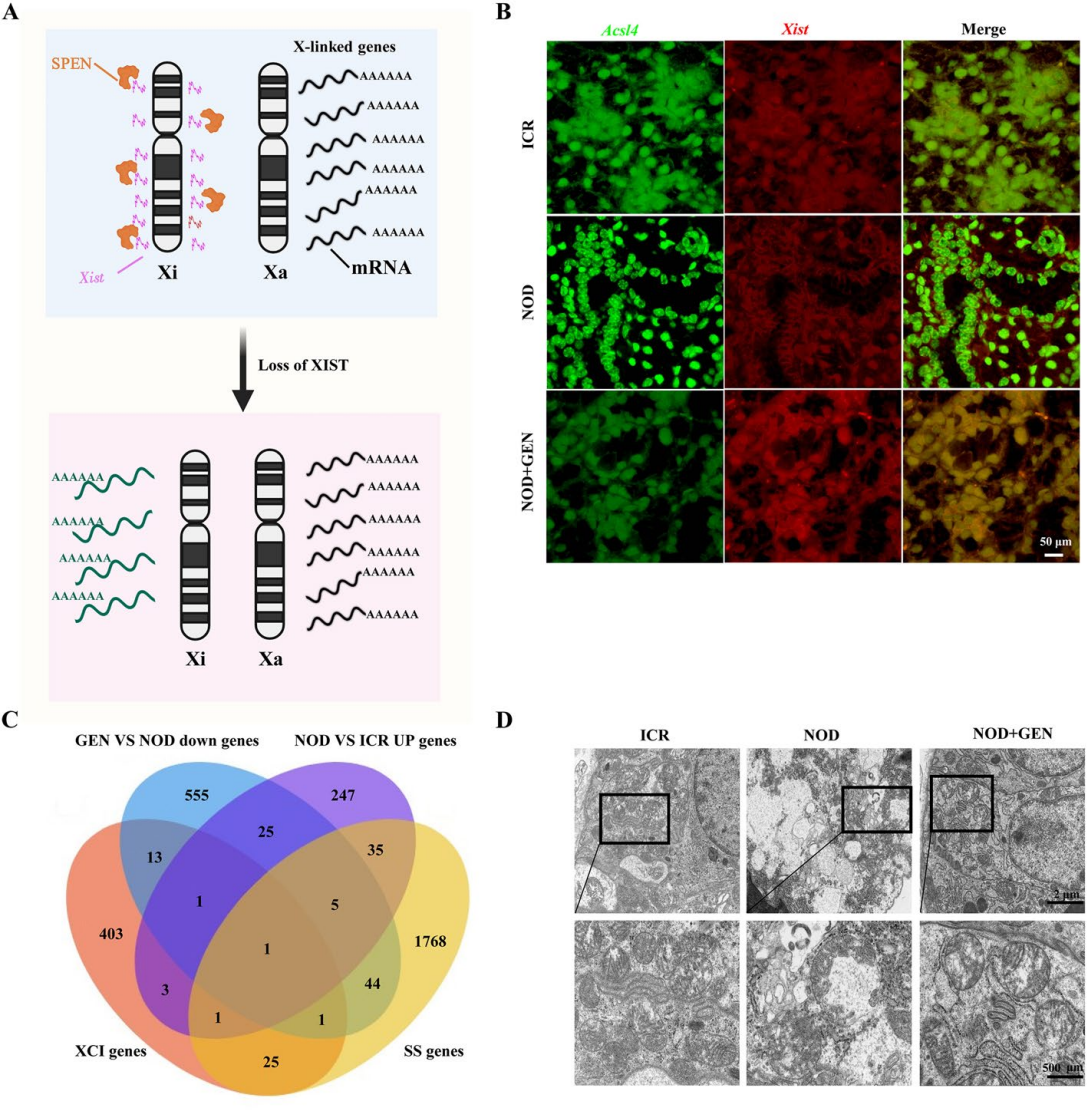
Genistein alleviated ferroptosis by *Xist*-mediated *Acsl4* gene silencing. **A** A schematic illustration of the molecular mechanism of *Xist*-mediating X-chromosome inactivation (XCI) via SPEN. **B** Venn analysis among XCI genes, SS genes, downregulated genes in genistein-treated mice, and upregulated genes in NOD/LtJ mice compared with ICR mice. **C** Fluorescence in situ hybridization (FISH) for *Xist* (red) and *Acsl4* (green) mRNA colocalization in submandibular gland tissues from ICR, NOD/LtJ, and genistein-treated NOD/LtJ mice. **D** Representative TEM images of submandibular gland tissues from ICR, NOD/LtJ, and genistein-treated NOD/LtJ mice. Xi, X chromosome inactivation; Xa, X chromosome activation

*Xist* is known to mediate XCI with SPEN serving as a key orchestrator of this process [32, 33] (Fig. 3A). The Venn diagram (Fig. 3B) showed that *Acsl4* was the crucial gene involved in the interactions of upregulated genes in genistein-treated NOD/LtJ mice compared with genes in untreated NOD/LtJ mice, as well as in downregulated genes in NOD/LtJ mice compared with XCI and SS-related genes in ICR mice. To support this, we assessed ACSL4 mRNA and protein expression patterns in the submandibular glands of ICR, NOD/LtJ, and genistein-treated NOD/LtJ mice (Supplementary Fig. S4A and B). RNA FISH analysis of *Xist* and *Acsl4* indicated that genistein downregulates the expression of *Acsl4*, a well-known regulator of ferroptosis [34], by upregulating the expression of *Xist* (Fig. 3C). The transmission electron microscopy (TEM) images of the submandibular glands showed intact and linear or granular mitochondria with an integral bilayer membrane structure in ICR mice. Ruptured mitochondrial membranes, swollen mitochondria, and decreased mitochondrial cristae were observed in untreated NOD/LtJ mice, with genistein treatment partially restoring damaged mitochondrial morphology (Fig. 3D). Our results confirmed that genistein alleviates ferroptosis in the salivary glands of NOD/LTJ mice, likely by *Xist*-mediated *Acsl4* gene silencing.

### Genistein ameliorated IFNγ-induced inflammation and ferroptosis in SGECs

**Fig. 4:**
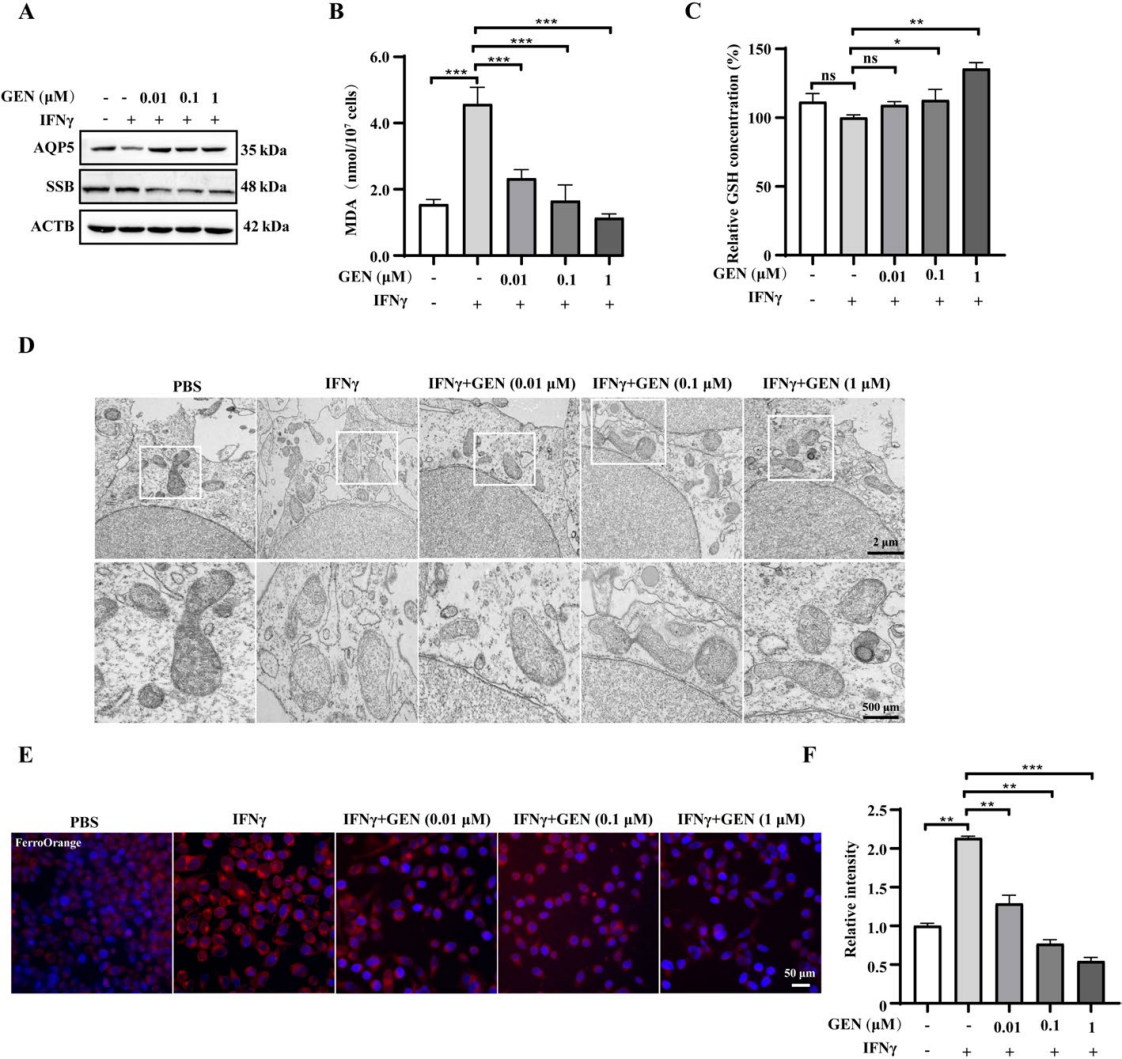
Genistein rescued IFNγ-induced inflammation and ferroptosis of SGECs. **A** Western blots of AQP5, and SSB proteins. MDA (**B**) and GSH (**C**) levels in SGEC lysates. **D** Representative TEM images of SGECs. **E**, **F** Representative images of FerroOrange staining. Significant differences between groups are indicated as: *p < 0.05, **p < 0.01, and ***p < 0.001; *n* = 3. ns, not significant; SGECs, salivary gland epithelial cells

To determine the optimum concentrations of genistein for SGECs treatment, we conducted CCK8 assays. Our results at the 24 h and 48 h time points indicated that a concentration of 1 μM genistein induced no significant cytotoxic effects on the cells (Supplementary Fig. S5A). IFNγ-treated SGECs were used to mimic inflammatory conditions and ferroptosis in SS [27]. Here, we observed a dose-dependent increase in the expression of AQP5 upon treatment with genistein in the IFN*γ*-treated SGECs (Fig. 4A and Supplementary Fig. S5B). Conversely, we found a dose-dependent decrease in the expression of SSB upon genistein treatment in IFNγ-treated SGECs (Fig. 4A and Supplementary Fig. S5B). In vivo, we observed ferroptosis in SS, with genistein improving damaged mitochondrial morphology.

To validate these findings, ferroptosis was examined in vitro. IFNγ-treated SGECs showed increased MDA levels and decreased GSH levels, with genistein treatment dose-dependently rectifying the effect of IFNγ on MDA and GSH production in SGECs (Fig. 4B, C). TEM images of the SGECs supported this finding (Fig. 4D). FerroOrange staining revealed higher Fe^2+^ levels in IFNγ-treated SGECs, and genistein treatment dose-dependently inhibited IFNγ-induced Fe^2+^ levels, consistent with our MDA, GSH, and TEM results (Fig. 4E, F). The effects of genistein on inflammation, ferroptosis, and function of IFNγ-SGECs in vitro were similar to in vivo results in NOD/LtJ mice.

### Genistein reduced inflammation and ferroptosis in SGECs by upregulated-*XIST*-mediated *ACSL4* gene silencing

**Fig. 5:**
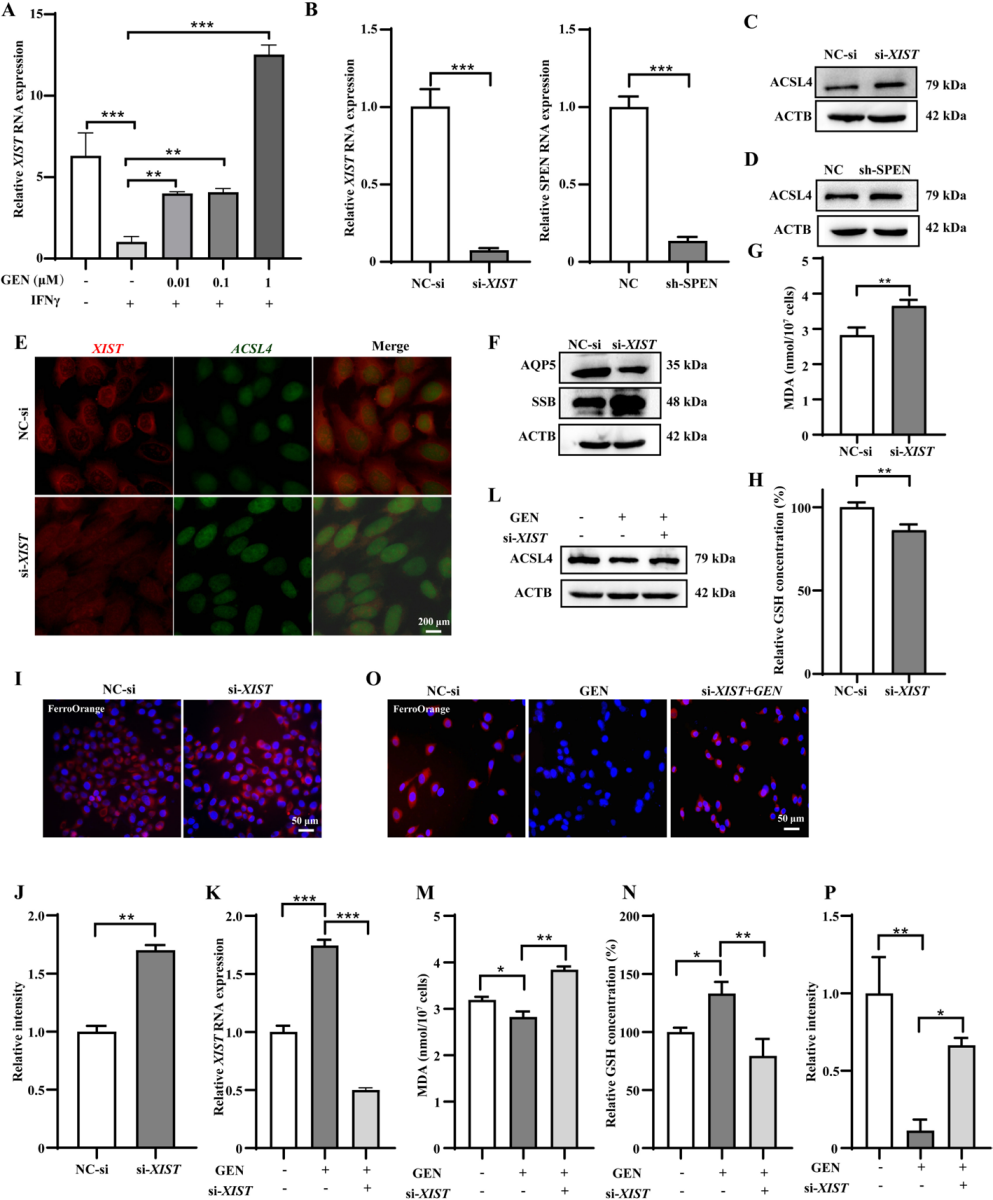
Genistein reduced inflammation and ferroptosis by upregulating *XIST*-mediated *ACSL4* gene silencing in SGECs. **A** The level of RNA expression of *XIST* in SGECs. **B** The transfection efficiency of si-*XIST* in SGECs by the RT-qPCR assay. **C**, **D** The expression levels of ACSL4 proteins evaluated by western blotting. **E** Fluorescence in situ hybridization (FISH) for *XIST* (red) and *ACSL4* (green) mRNA colocalization in SGECs. **F** Western blotting of AQP5, and SSB proteins in *XIST* knockdown SGECs. The MDA (**G**) and GSH (**H**) levels in *XIST* knockdown SGECs. **I**, **J** Representative images of FerroOrange staining in *XIST* knockdown SGECs. **K** The *XIST* expression pattern in genistein-treated *XIST*-knockdown SGECs. **L** Western blotting of ACSL4 proteins in genistein-treated *XIST*-knockdown SGECs. The MDA (**M**) and GSH levels (**N**) in genistein-treated *XIST*-knockdown SGECs. **O**, **P** Representative images of FerroOrange staining in genistein-treated *XIST*-knockdown SGECs. Significant differences are indicated as: *p < 0.05, **p < 0.01, and ***p < 0.001; *n* = 3

*Xist* was upregulated in SGECs of genistein-treated NOD/LtJ mice (Fig. 2). We initially found that RNA levels of *XIST* were downregulated in IFNγ-treated SGECs, and genistein effectively corrected this effect in a dose-dependent manner (Fig. 5A). SPEN is an important protein that combines with *XIST* to facilitate its role in XCI. Both *XIST* knockdown (Fig. 5B,C and Supplementary Fig. S5C) and SPEN knockdown (Fig. 5B,D and Supplementary Fig. S5D) upregulate ACSL4 protein expression in SGECs. RNA-FISH analysis confirmed *ACSL4* upregulation in SGECs with *XIST* knockdown (Fig. 5E). These results further validate our finding that *XIST* suppresses ACSL4 via XCI. *XIST* knockdown downregulated AQP5 and occludin expression and upregulates SSB expression in SGECs (Fig. 5F and Supplementary Fig. S5E). *XIST* knockdown increased MDA levels and decreased GSH levels in SGECs (Fig. 5G, H).

FerroOrange staining showed higher Fe^2+^ levels in *XIST*-knockdowned SGECs (Fig. 5I, J), indicating that *XIST* plays a regulatory role in ferroptosis. Genistein failed to downregulate ACSL4 expression in *XIST*-knockdown SGECs (Fig. 5K, L and Supplementary Fig. S5F). Similarly, genistein did not upregulate MDA levels and downregulate GSH and Fe^2+^ levels in *XIST*-knockdowned SGECs (Fig. 5M–P). These results revealed that genistein reduces inflammation, ferroptosis, and functions of SGECs by upregulating *XIST*-mediated *ACSL4* gene silencing.

### Genistein downregulated ACSL4 to reduce inflammation and ferroptosis

**Fig. 6:**
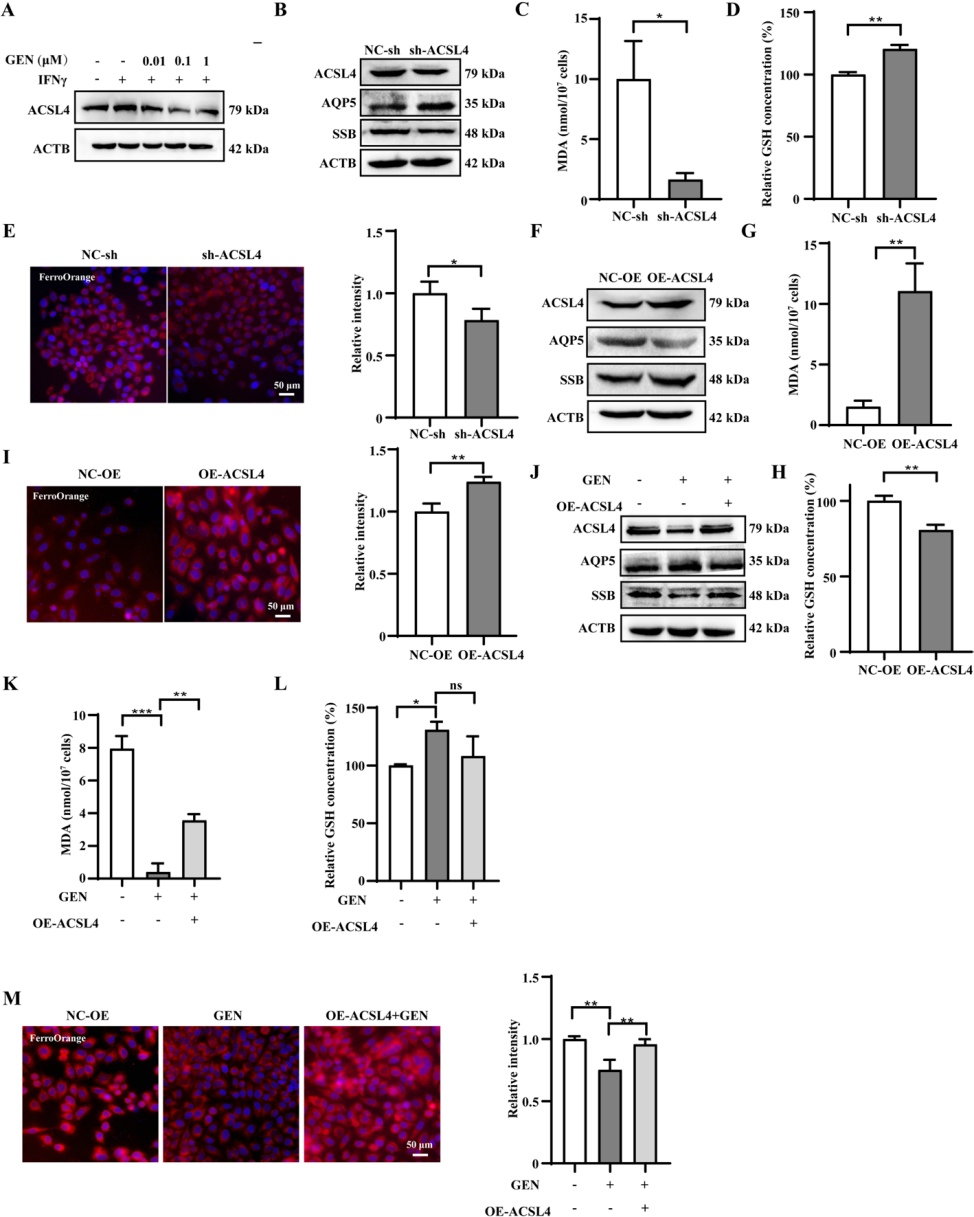
Genistein downregulated ACSL4 to reduce inflammation and ferroptosis in SGECs. **A** Western blots of ACSL4. **B** Western blots of AQP5, SSB, and ACSL4 proteins in ACSL4-knockdown SGECs. The MDA (**C**) and GSH (**D**) levels in ACSL4-knockdown SGECs. **E** Representative images of FerroOrange staining in ACSL4-knockdown SGECs. **F** Western blots of AQP5, SSB, and ACSL4 proteins in ACSL4-overexpression SGECs. MDA (**G**) and GSH (**H**) levels in ACSL4-overexpression SGECs. **I** Representative images of FerroOrange staining ACSL4-overexpression SGECs. **J** Western blots of AQP5, SSB, and ACSL4 proteins in genistein-treated ACSL4-overexpression SGECs. MDA (**K**) and GSH (**L**) levels in genistein-treated ACSL4-overexpression SGECs. **M** Representative images of FerroOrange staining and quantification in genistein-treated ACSL4-overexpression SGECs. Significant differences are indicated as: *p < 0.05, **p < 0.01, and ***p < 0.001, ns, not significant; *n* = 3

ACSL4 was upregulated in IFNγ-treated SGECs, but genistein reversed this effect in a dose-dependent manner (Fig. 6A and Supplementary Fig. S6A). Our results showed that expressions of ACSL4, and SSB were downregulated, whereas expressions of AQP5 and occludin were upregulated in *ACSL4* knockdown SGECs (Fig. 6B and Fig. S6B). *ACSL4* knockdown SGECs show decreased MDA levels and increased GSH levels (Fig. 6C, D). FerroOrange staining revealed lower levels of Fe^2+^ in *ACSL4* knockdown SGECs (Fig. 6E). Moreover, our results showed that expression of ACSL4, and SSB were upregulated, and expression of AQP5 was downregulated in ACSL4-overexpressed SGECs (Fig. 6F and Supplementary Fig. S6C).

*ACSL4* overexpressed SGECs showed increased MDA levels and decreased GSH levels (Fig. 6G, H). FerroOrange staining revealed higher levels of Fe^2+^ in *ACSL4* overexpressed SGECs (Fig. 6I). Genistein failed to upregulate APQ5 and downregulate SSB in *ACSL4* overexpressed SGECs (Fig. 6J and Supplementary Fig. S6D). These results indicate that genistein alleviates SS symptoms by downregulating ACSL4. Similarly, genistein did not reduce ferroptosis in *ACSL4* overexpressed SGECs (Fig. 6K–M). Overall, these results demonstrated that genistein reduces inflammation, ferroptosis, and the function of SGECs by downregulating ACSL4.

## Discussion

Sjögren’s syndrome predominantly affects women with a female-to-male ratio of 14:1 [6]. However, the biological basis remains largely unexplained. Salivary glands are known to express ERα, suggesting that the decline of E2 in postmenopausal women directly increases the risk of SS [35]. Studies show that hormone replacement therapy increases the flow rate significantly in postmenopausal women [36–38]. In this study, we confirmed that genistein binds to ERα directly using in silico methods. Recent research suggested that, beyond the declined level of E2, X chromosome genes are also responsible for female predisposition to SS [39]. In this study, the *Xist* gene was downregulated under SS conditions and upregulated following genistein treatment. More surprisingly, the genistein_ERα complex upregulates *Xist* expression by binding to the estrogen response elements region of *Xist*.

Reduced E2 levels and disrupted E2/ERα signaling in SGECs are commonly observed in SS [40, 41]. In this study, we found that, among various flavonoids, genistein exhibits the strongest binding affinity to ERα. We further found that genistein attenuates the SS-like phenotype, characterized by a reduced number of infiltrated lymphocytes, increased saliva secretion, and decreased water intake. Using mRNA sequencing and bioinformatics analyses, we revealed that genistein mechanistically upregulates *Xist* expression in SGECs. In females, *XIST* attenuates inflammatory response [42], and its dysregulation in B or T cells contributes to female-biased autoimmunity [43, 44]. However, the role of *XIST* in SGEC viability and function during SS remains unknown. Here, we discovered that *XIST* is downregulated in SGECs during SS and that *XIST*-knockout in SGECs induces SS-like phenotypes, characterized by decreased AQP5 expression, and increased SSB antigen levels.

We validated that genistein upregulates the *XIST* gene in SGECs via the ERα signaling pathway. *XIST* is the trigger and master regulator of XCI [45]. We found that the XCI gene-ACSL4 is upregulated in SS and downregulated following genistein treatment. When *XIST* was knocked out in SGECs, ACSL4 expression increased, and genistein failed to downregulate the expression of ACSL4, suggesting that ASCL4 is a downstream target of *XIST*. These findings provide a link between *XIST* expression and ferroptosis in SS. Therefore, genistein binding to ERα upregulates *XIST* to attenuate the SS-like phenotype.

The ACSL4 signaling pathway is known to contribute to cell death by inducing ferroptosis [46, 47]. Our previous study shows that ferroptosis is a potential pathway in the pathology of SS [23]. However, the regulatory role of ASCL4 on the SS phenotype in SGECs remained unknown. Our present results showed that ACSL4 was upregulated in SS and downregulated upon genistein treatment. ACSL4 knockout could alleviate the SS-like phenotype and ferroptosis, whereas ACSL4 overexpression triggers SS in SGECs. Genistein failed to alleviate the SS-like phenotype and ferroptosis in SGECs following ACSL4 overexpression. Our findings indicate that genistein attenuates the SS-like phenotype and ferroptosis through *XIST* upregulation followed by ACSL4 downregulation.

Nonetheless, this study does have some limitations. Firstly, our study only concentrated on the effects of genistein on SGECs, without assessing its influence on immune cells such as acinar B/T cells. Secondly, we did not provide direct evidence of how *Xist* inhibits ACSL4 expression. Thirdly, further comprehensive investigations are warranted to delve into the role of *Xist* in SS development and progression in *Xist*-knockout mice.

## Conclusions

Genistein has a significant therapeutic effect in the SS mouse model. It can restore the pathological damage in salivary gland tissue and reverse the ferroptosis of SGECs. Genistein increases the target gene *Xist* expression by binding to ERα, and subsequently decreasing the XCI-gene ACSL4 expression, thereby inhibiting ferroptosis. This supports future preclinical and clinical treatment for SS using genistein. *Xist* upregulation in SGECs offers a novel clinical therapeutic target and approach for SS.

## Supplementary Information

Additional file 1.

Additional file 2.

## Data Availability

All datasets used or analyzed in this study are available from the corresponding author upon request.

## Abbreviations

ACTB: β-Actin
ACSL4: Acyl-CoA synthetase long-chain family member 4
AQP5: Aquaporin 5
CCK-8: Cell counting kit-8
CHIP: Chromatin immunoprecipitation
DAB: Diaminobenzidine
DEGs: Differentially expressed genes
EGCG: Epigallocatechin-3-gallate
ELISA: Enzyme-linked immunosorbent assay
ERα: Estrogen receptor-alpha
FLS: Focal lymphocytic sialadenitis
GO: Gene ontology
GSH: Glutathione
H&E: Hematoxylin–eosin
ICR: Institute of Cancer Research
IHC: Immunohistochemistry
IFNγ: Interferon-γ
IL: Interleukin
JAK2: Janus kinase 2
KEGG: Kyoto Encyclopedia of Genes and Genomes
MD: Molecular dynamics
MDA: Malondialdehyde
NOD: Nondiabetic obese
OE RNA: RNA overexpression
RMA: Robust multiarray mean algorithm
RNA-FISH: RNA-fluorescence in situ hybridization
RT-qPCR: Real-time quantitative polymerase chain reaction
SGECs: Salivary gland epithelial cells
shRNA: Short hairpin RNA
siRNA: Short interfering RNA
SPR: Surface plasmon resonance
SS: Sjögren’s syndrome
SSB: Sjögren’s syndrome antigen B (autoantigen La)
STAT3: Signal transducer and activator of transcription 3
TEM: Transmission electron microscopy
VEGF: Vascular endothelial growth factor
XCI: X chromosome inactivation
Xi: Inactivated X
*Xist*: X-inactive specific transcript
