# Supplementary material for: Salivary gland protective and antiinflammatory effects of genistein in Sjögren’s syndrome by inhibiting Xist/ACSL4-mediated ferroptosis following binding to estrogen receptor-alpha
Source: Cell Mol Biol Lett. 2024 Dec 2;29:147. doi: 10.1186/s11658-024-00667-6 (PMC11613825; doi:10.1186/s11658-024-00667-6)
Supplement: Supplementary file 1 — Additional file 1. [file 11658_2024_667_MOESM1_ESM.pdf]

The triplicates for each western blot in manuscript

Salivary gland protective and anti-inflammation effects of genistein in Sjögren's syndrome by inhibiting Xist/ACSL4-mediated ferroptosis following binding to estrogen receptor-alpha

The triplicates for each western blot in **Figure 4A**

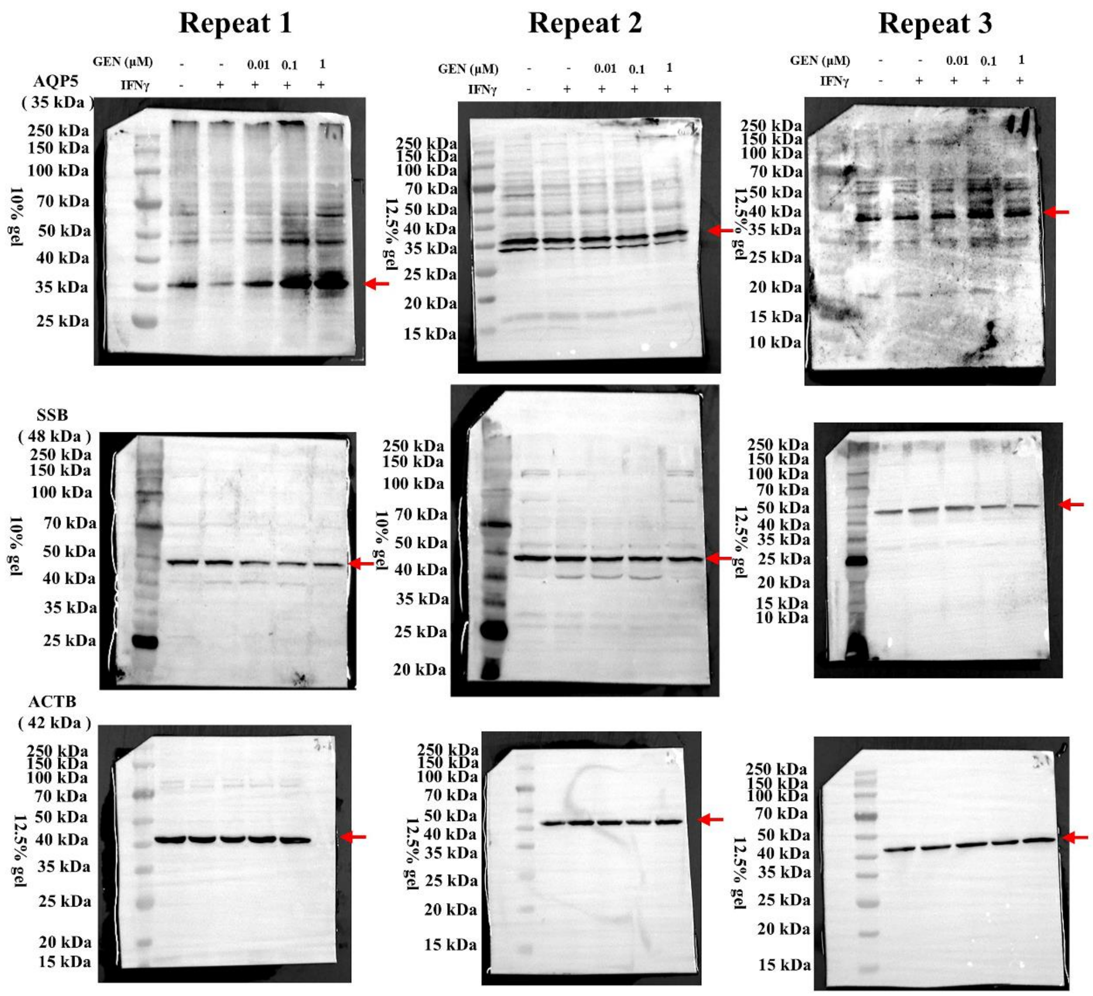

Fig S1 The western blots of AQP5, and SSB proteins.

The triplicates for each western blot in **Figure 5C**

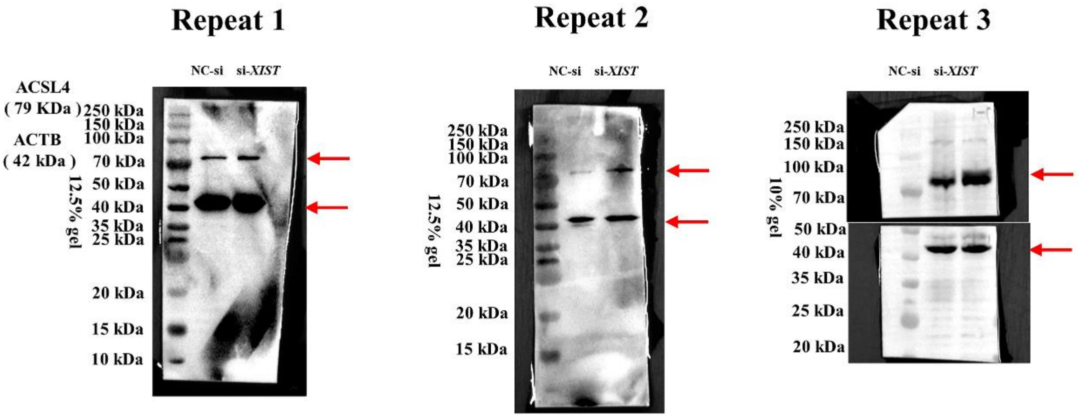

Fig S2 The expression levels of ACSL4 proteins in *XIST* knockdown SGECs were evaluated by western blotting

The triplicates for each western blot in **Figure 5D**

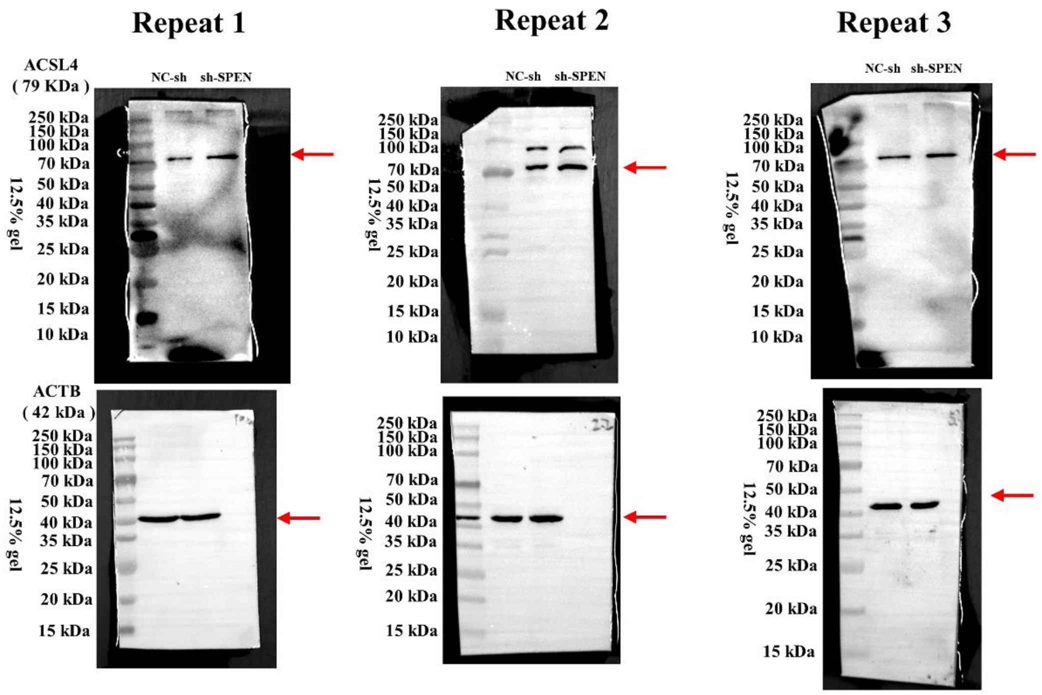

Fig S3 The expression levels of ACSL4 proteins in SPEN knockdown SGECs were evaluated by western blotting

The triplicates for each western blot in **Figure 5F**

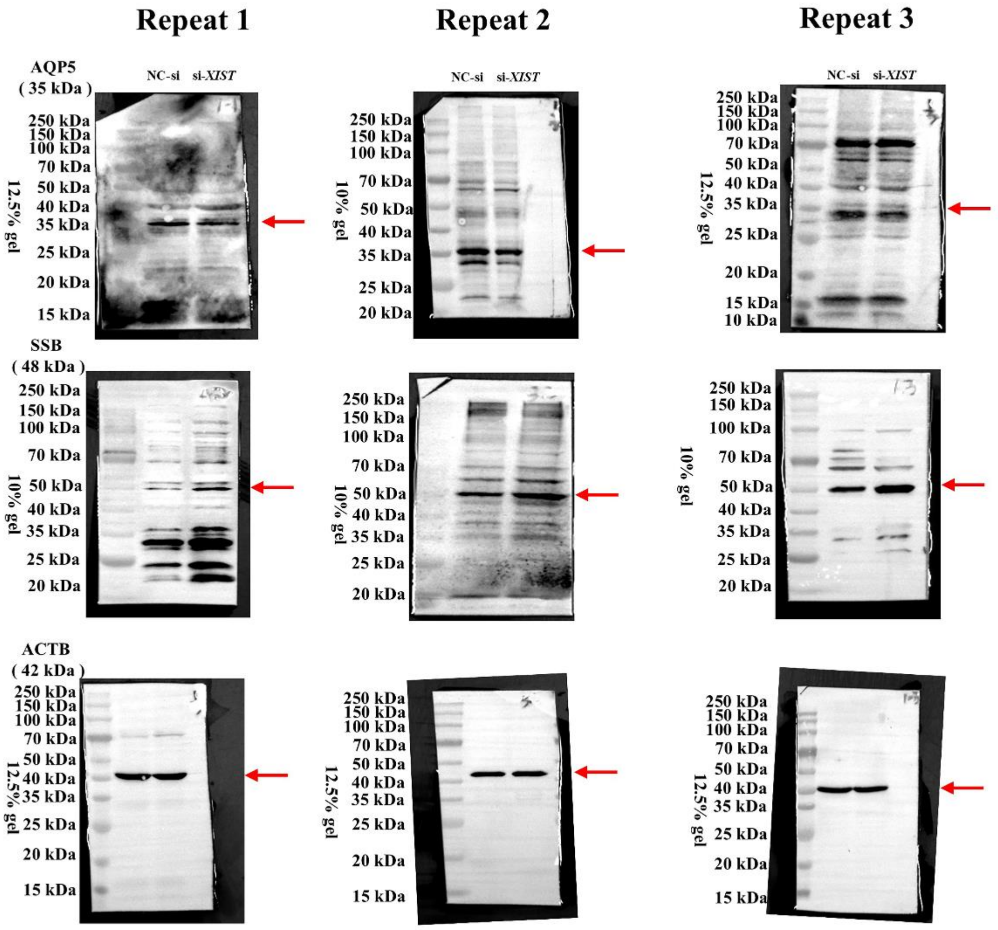

Fig S4 Western blotting of AQP5, and SSB proteins in *XIST* knockdown SGECs.

The triplicates for each western blot in **Figure 5L**

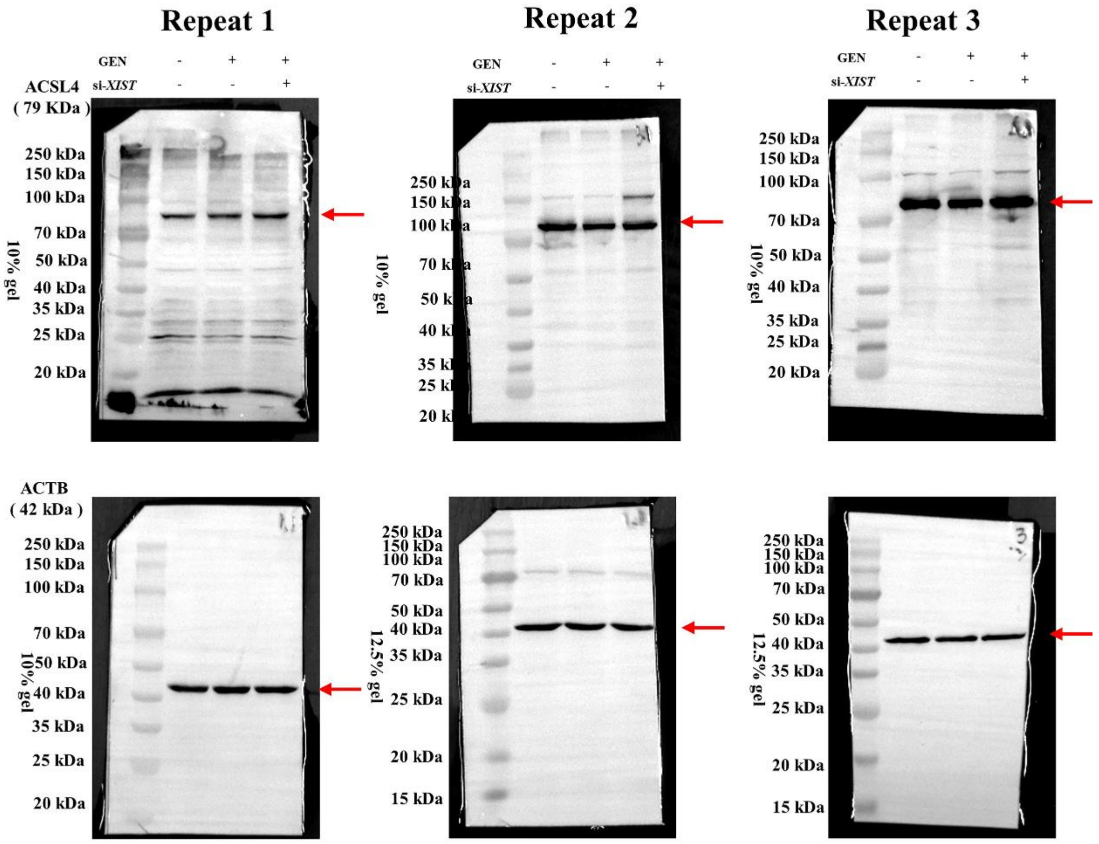

Fig S5 Western blotting of ACSL4 proteins in genistein-treated *XIST*-knock down SGECs.

The triplicates for each western blot in **Figure 6A**

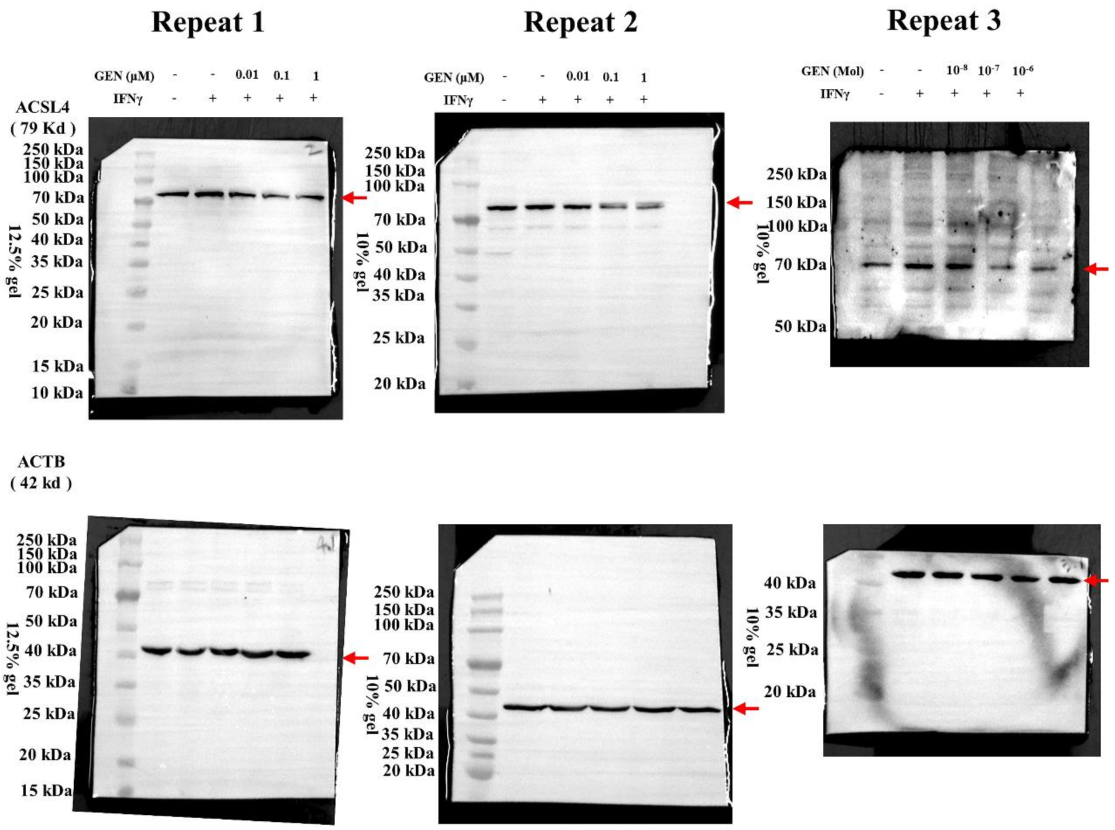

Fig S6 Western blots of ACSL4.

The triplicates for each western blot in **Figure 6B**

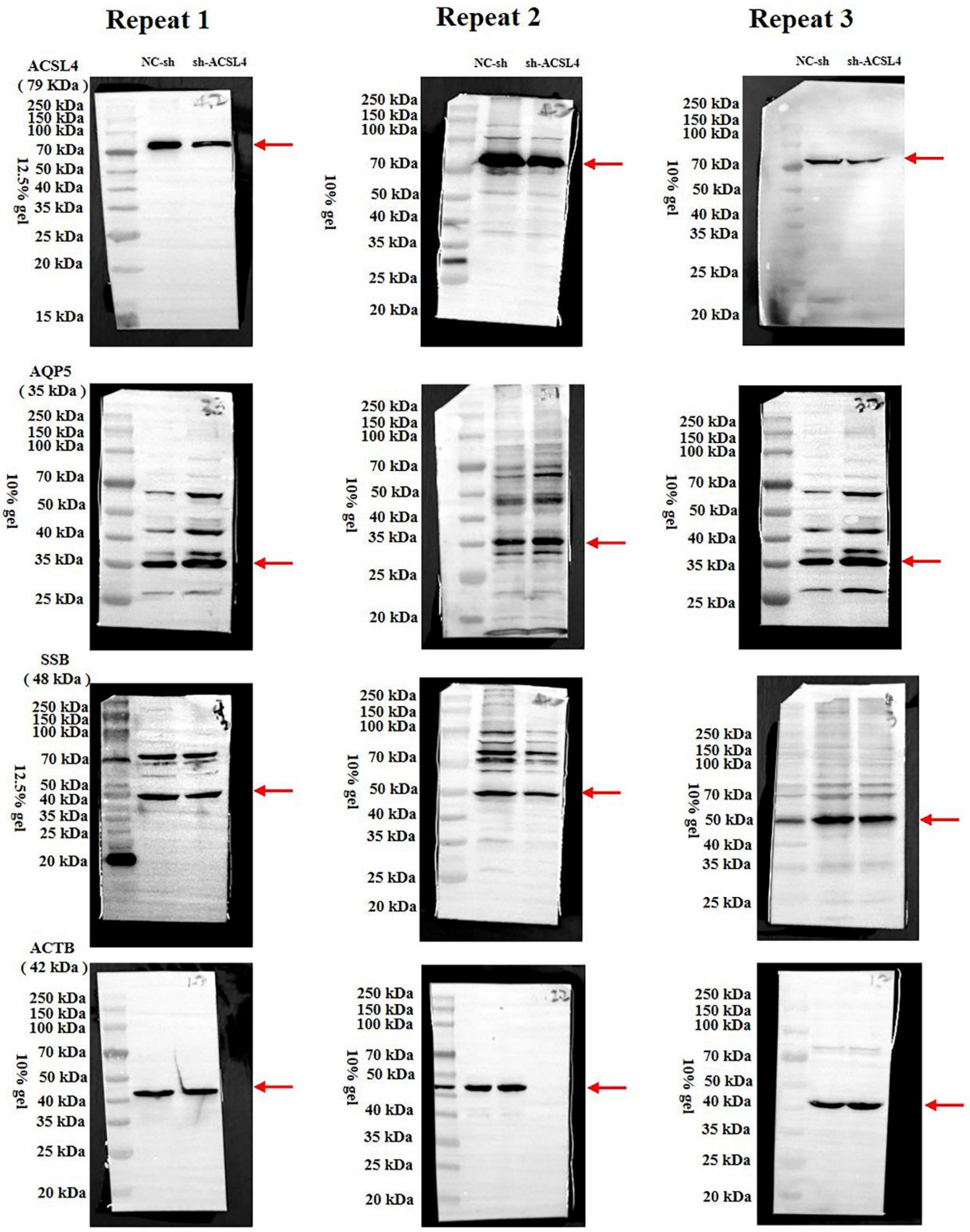

Fig S7 Western blots of AQP5, SSB, and ACSL4 proteins in ACSL4-knockdown SGECs

The triplicates for each western blot in **Figure 6F**

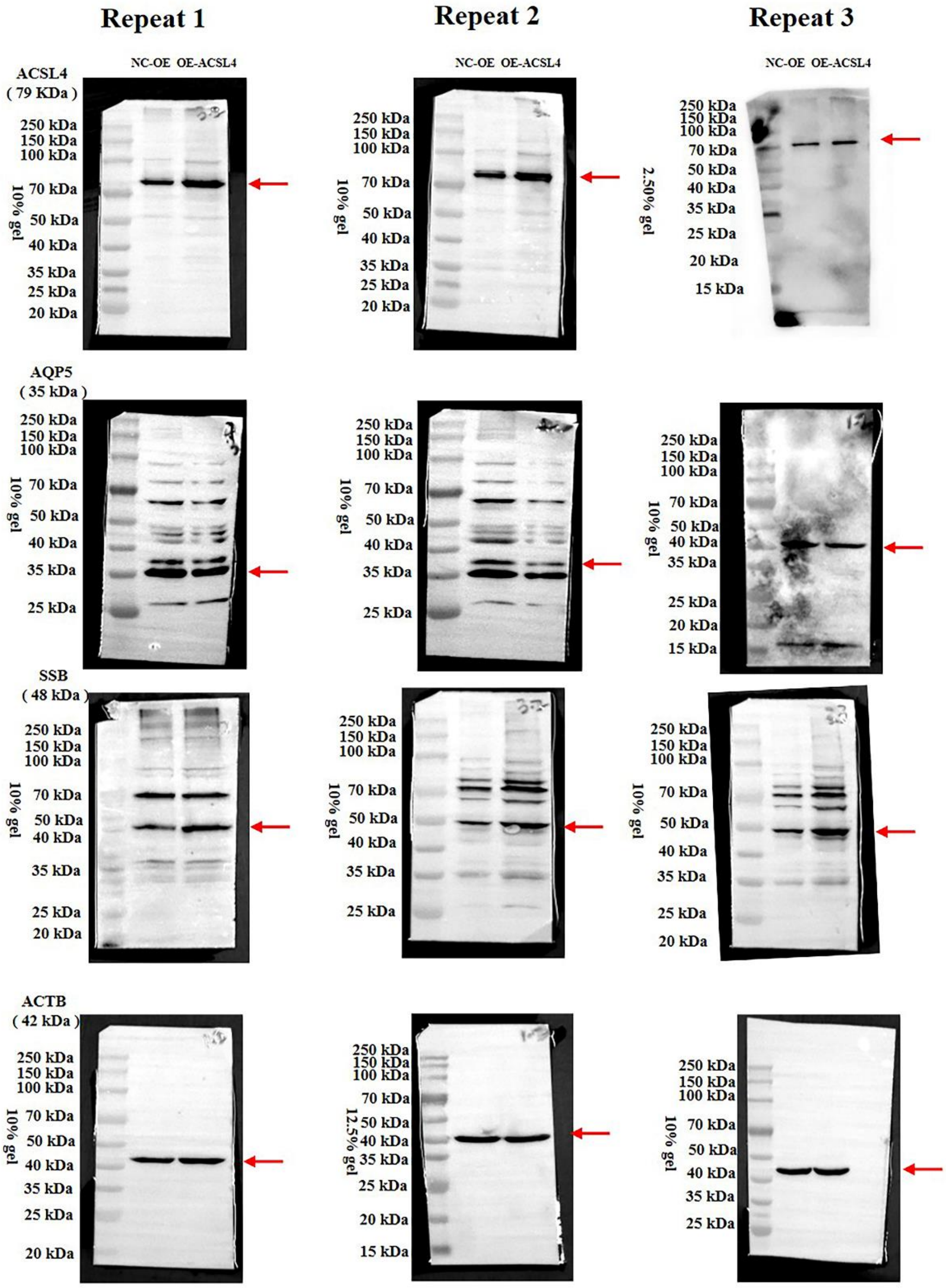

Fig S8 Western blots of AQP5, SSB, and ACSL4 proteins in ACSL4-overexpression SGECs.

The triplicates for each western blot in **Figure 6J**

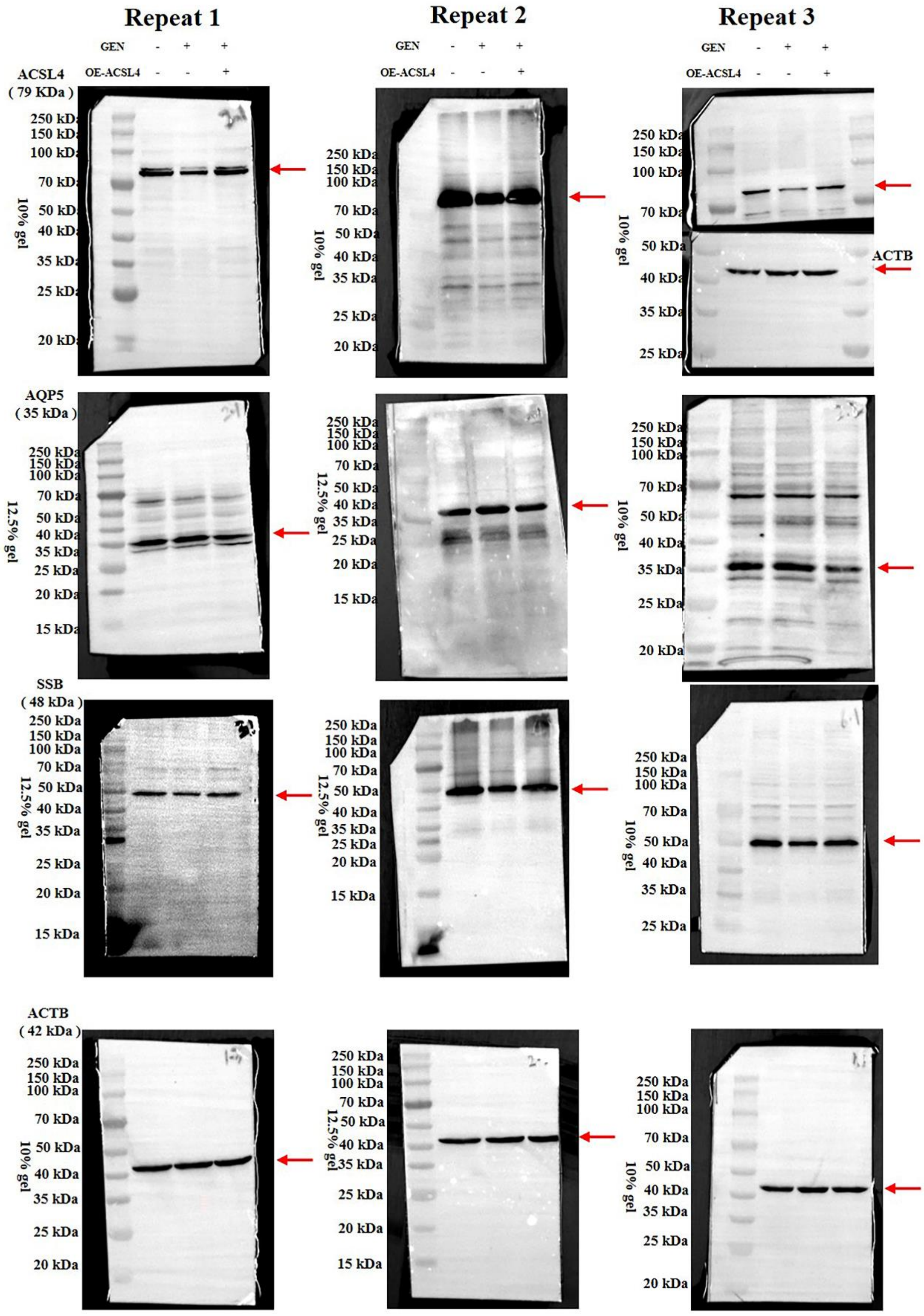

Fig S9 Western blots of AQP5, SSB, and ACSL4 proteins in genistein-treated ACSL4-overexpression SGECs.
