# Supplementary material for: Salivary gland protective and antiinflammatory effects of genistein in Sjögren’s syndrome by inhibiting Xist/ACSL4-mediated ferroptosis following binding to estrogen receptor-alpha
Source: Cell Mol Biol Lett. 2024 Dec 2;29:147. doi: 10.1186/s11658-024-00667-6 (PMC11613825; doi:10.1186/s11658-024-00667-6)
Supplement: Supplementary file 2 — Additional file 2. [file 11658_2024_667_MOESM2_ESM.docx]

**Supporting Information for Original article**

**Supporting figures**


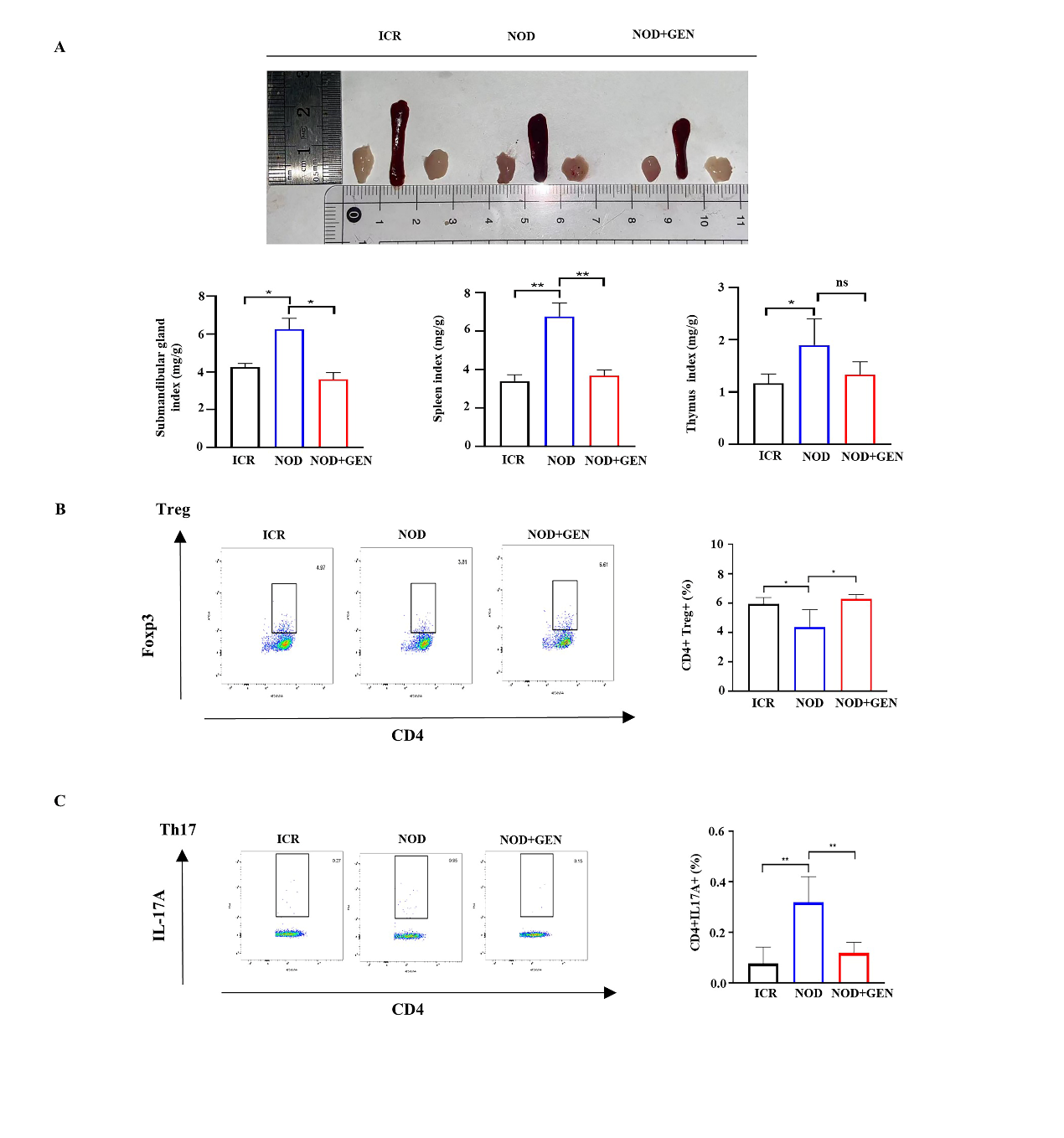


**Fig S1.** Treatment of genistein alleviates organ inflammation in mice. (A) The submandibular gland index, spleen index, and thymus index in mice. (B) the Treg and Th 17 cells in mice spleen. The significant difference between the groups: * *p* < 0.05, ** *p* < 0.01, and *** *p* < 0.001, ns, statistically not significant; n = 5.


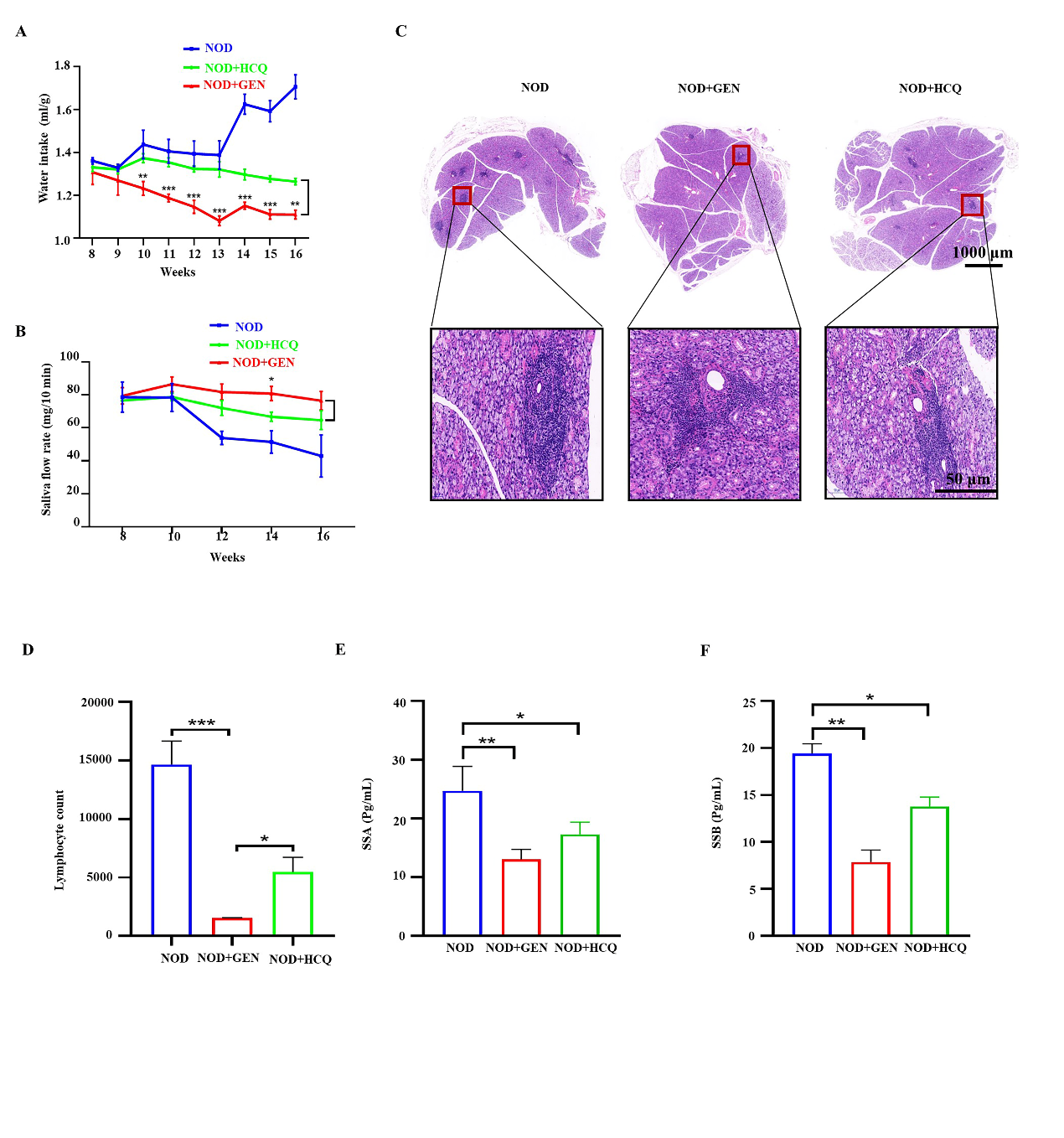


**Fig S2.** Treatment of hydroxychloroquine (HCQ) alleviates Sjögren’s syndrome features in NOD mice. (A, B) Hematoxylin-eosin (H&E) stained images and lymphocyte infiltration count in mice submandibular gland tissues. (C) Assessment of water intake of experimental animals. (D) Assessment of salivary flow rate of experimental animals. (E) The anti-SSA levels in NOD/LtJ mice serum. (F) The anti-SSB levels in NOD/LtJ mice serum. Significant difference between the groups: * *p* < 0.05, ** *p* < 0.01, and *** *p* < 0.001; n = 5.


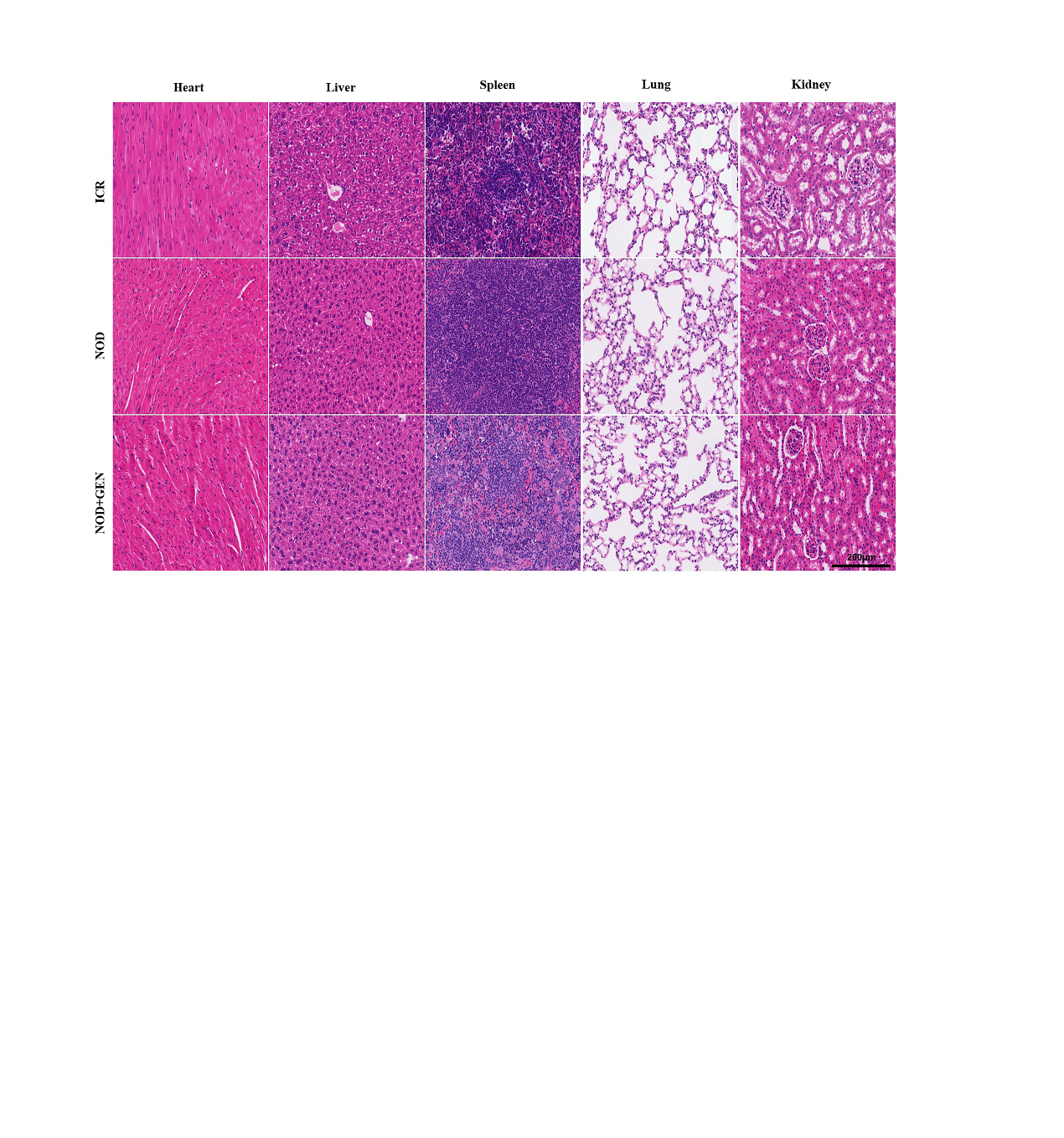


**Fig S3.** The H&E imaging of representative organs, including the heart, liver, spleen, lung, and kidney.


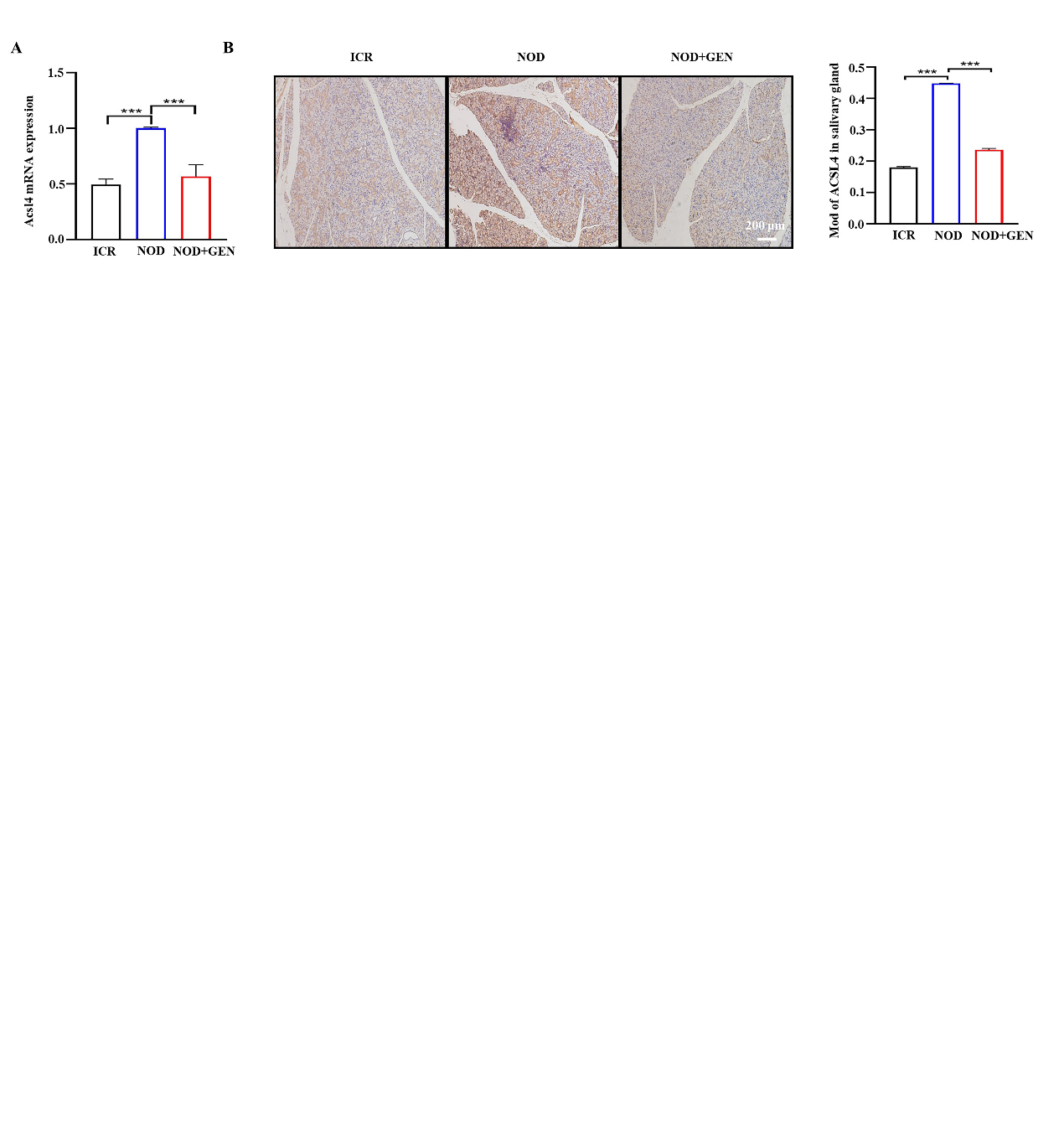


**Fig S4.** The expression of ACSL4 in submandibular gland tissues. (A) The mRNA expression of *Acsl4* in submandibular gland tissues. (B) Immunohistochemistry images and analysis of ACSL4 of submandibular gland tissues. Significant difference between the groups: * *p* < 0.05, ** *p* < 0.01, and *** *p* < 0.001; n = 5.


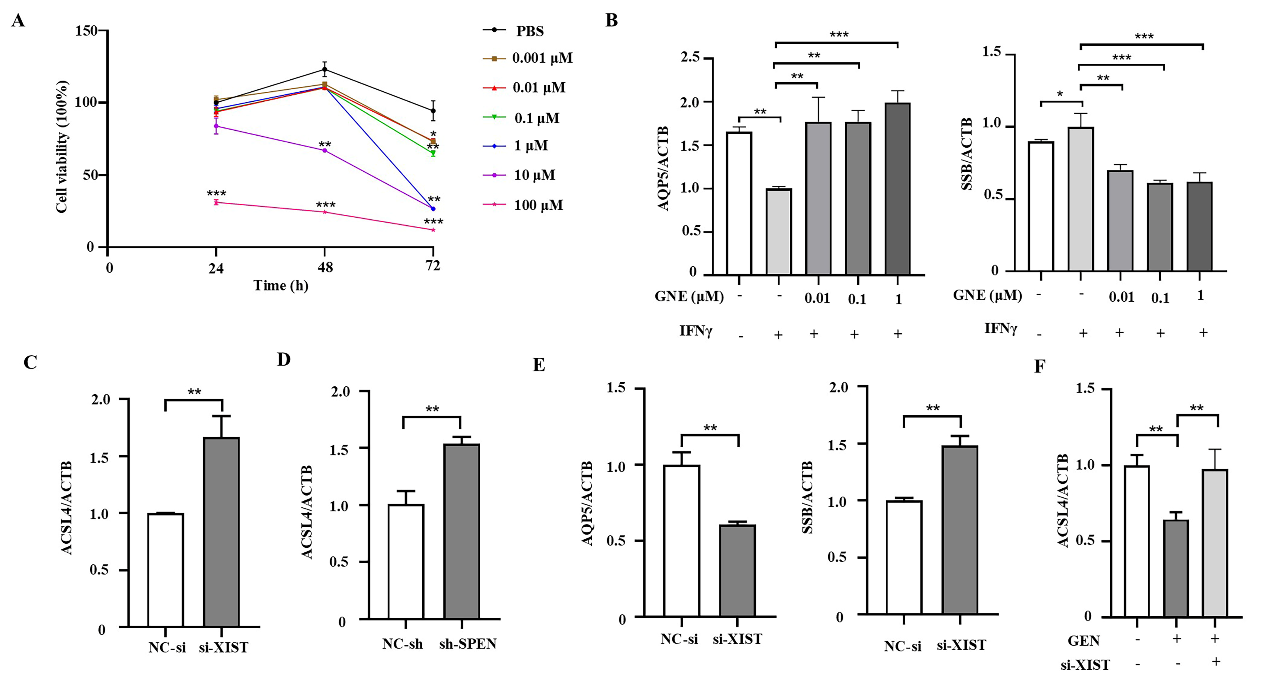


**Fig S5.** (A) Relative viabilities of SGECs treated with genistein at different concentrations. Western blot analysis. (B) Quantification of AQP5, and SSB proteins from western blot. (C) Quantification of ACSL4 protein transfected with Si-XIST from western blot. (D) Quantification of ACSL4 protein transfected with Sh-SPEN from western blot. (E) Quantification of AQP5, and SSB proteins transfected with Si-XIST from western blot. (F) Quantification of ACL4 proteins transfected with Si-XIST or treatment with genistein from western blot. Significant difference between the groups: * *p* < 0.05, ** *p* < 0.01, and *** *p* < 0.001; n = 3.


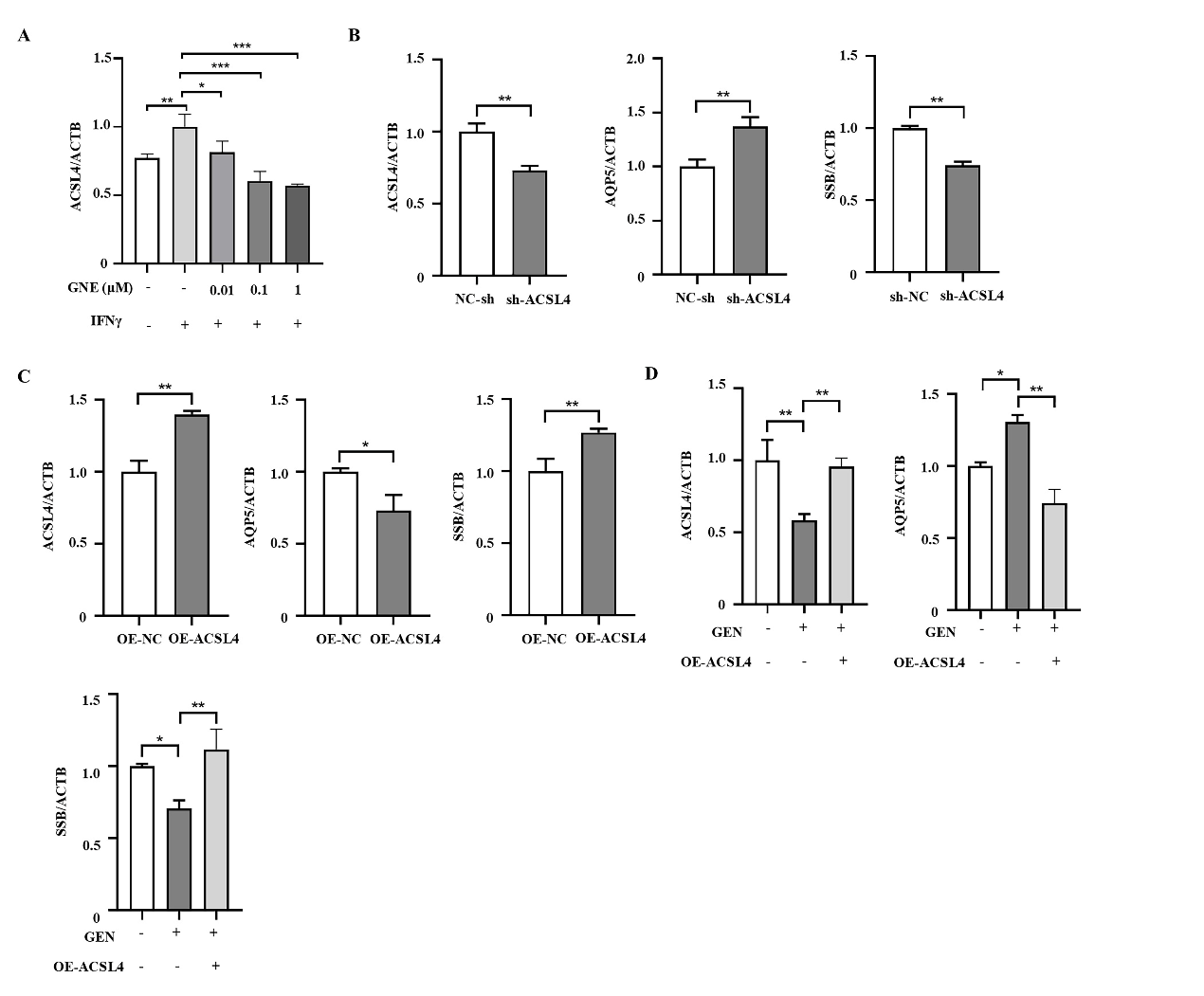


**Fig S6.** Western blot analysis. (A) Quantification of ACSL4 protein treated with IFN*γ* or genistein from western blot. (B) Quantification of AQP5, SSB, and ACL4 proteins transfected with Sh-ACSL4 from western blot. (C) Quantification of AQP5, SSB, and ACL4 proteins transfected with OE-ACSL4 from western blot. (D) Quantification of AQP5, SSB, and ACL4 proteins transfected with OE-ACSL4 or treatment with genistein from western blot. Significant difference between the groups: * *p* < 0.05, ** *p* < 0.01, and *** *p* < 0.001; n = 3.

**Supporting tables**

**Table S1.** PCR primers used in this study

| Gene | Forward sequence (5'->3') | Reverse sequence (5'->3') | Product length (bp) |
| --- | --- | --- | --- |
| *Acsl4* | TCCTCCAAGTAGACCAACCCC | AGTCCAGGGATACGTTCACAC | 141 |
| *Xist* | ATTCGCCTTGATTTGTGG | TAGTCCTCTGCGGCTTCC | 108 |
| *Actb* | AACAGTCCGCCTAGAAGCAC | CGTTGACATCCGTAAAGACC | 281 |
| *Aqp5* | CCGTGGTGGTGGAGTTAATCTTGAC | TGTGACCGACAAGCCAATGGATAAG | 119 |
| *Cxcl10* | ATCCACCGCTGAGAGACATCCC | AATGACGGCAGCACTTGGGTTC | 80 |
| *Cxcl13* | ACTCCACCTCCAGGCAGAATGAG | TGTGTAATGGGCTTCCAGAATACCG | 102 |
| *Il1β* | GAAATGCCACCTTTTGACAGTG | TGGATGCTCTCATCAGGACAG | 116 |
| *Il6* | TAGTCCTTCCTACCCCAATTTCC | TTGGTCCTTAGCCACTCCTTC | 76 |
| *ACSL4* | CATCCCTGGAGCAGATACTCT | TCACTTAGGATTTCCCTGGTCC | 96 |
| *SPEN* | GAGTATCGTGGAGAAGCCCG | TCGGGATGTTCAAGGTCTGC | 174 |
| *XIST* | AGTGTACCTACCGCTTTGG | GACTTCCTCTGCCTGACC | 180 |
| *ACTB* | ATCACTATTGGCAACGAGCGGTTC | CAGCACTGTGTTGGCATAGAGGTC | 156 |
| *AQP5* | TCCATGAACCCAGCCCGATCTT | GAAGTAGAGGATTGCAGCCAGG | 200 |

**Table S2**. Sequences of probes used in this study

| Type | Sequences (5'->3') |
| --- | --- |
| *Xist* (mouse) | agcaataggaccgtatgcaatccaatgcttaggaagagggaaagaatgcggccttgttga |
| *Acsl4* (mouse) | cttcttcccaaacttggataccatgaactactgcttcttttctcaaatccttcagggtac |
| *XIST* (human) | gaatcagcaggtatccgatactgtgctacagactcttcaacctaacttcaacgtaccgaggctgaatgcaaatggggttc |
| *ACSL4* (human) | cttcttcccaaacttggataccatgaactactgcttcttttctcaaatccttcagggtac |

**Table S3.** ChIP-qPCR primers for specific *XIST* promotor region amplification

| Biding site (BS) | Primer Sequence |
| --- | --- |
| h*XIST*-ERE1 | F: TACCCCAGGTGCATTTTTAATCT  R: TCCATTCCTGTACACTTAACTAGG |
| h*XIST*-ERE2 | F: TAGTTAAGTGTACAGGAATGGAGGT  R: AGCATGAAGGGATATGTGCTAA |

**Table S4**. Sequences of siRNA and shRNA

| Type | sense | antisense |
| --- | --- | --- |
| Control si*XIST* | UUCUCCGAACGUGUCACGUTT | ACGUGACACGUUCGGAGAATT |
| Si *XIST* | GCAUGCAUCUUGGACAUUUTT | AAAUGUCCAAGAUGCAUGCTT |
| Control shSPEN | CCTAAGGTTAAGTCGCCCTCG | CGAGGGCGACTTAACCTTAGG |
| Sh SPEN | CCCGTGGATATGGTTCAACTT | AAGTTGAACCATATCCACGGG |
| Control shACSL4 | GTTCTCCGAACGTGTCACGTTTCA | TTCAAGAGGCTTGCACAGTGCAAGAG |
| ShACSL4 | GCAGTAGTTCATGGGCTAAATTTCA | TTCGTCATCAAGTACCCGATTTAAGAG |

**Table S5**. Flavonoids compounds with ERα

| Rank | Name | CAS | Binding enegies (Kcal/mol) |
| --- | --- | --- | --- |
| 1 | Genistin | 529-59-9 | -10.452 |
| 2 | Poncirin | 14941-08-3 | -10.43 |
| 3 | Hesperidin | 520-26-3 | -10.37 |
| 4 | Grosvenorine | 156980-60-8 | -10.22 |
| 5 | Epimedin A1 | 140147-77-9 | -10.15 |
| 6 | Nepitrin | 569-90-4 | -9.996 |
| 7 | Rottlerin | 30169 | -9.924 |
| 8 | Clitorin | 55804-74-5 | -9.841 |
| 9 | Quercetin 3-O-beta-D-glucose-7-O-beta-D-gentiobioside | 60778-02-1 | -9.727 |
| 10 | Vitexin-4''-O-glucoside | 178468-00-3 | -9.585 |
| 11 | 4'-O-Glucosylvitexin | 76135-82-5 | -9.549 |
| 12 | Procyanidin B2 | 29106-49-8 | -9.493 |
| 13 | Bilobetin | 521-32-4 | -9.49 |
| 14 | Kuwanon H | 76472-87-2 | -9.476 |
| 15 | Loureirin D | 119425-91-1 | -9.439 |
| 16 | Puerarin-4'-O-D-glucopyranoside | 117047-08-2 | -9.431 |
| 17 | Silibinin | 22888-70-6 | -9.397 |
| 18 | 4',5-Dihydroxyflavone | 6665-67-4 | -9.337 |
| 19 | Vitexin-2'-O-rhamnoside | 64820-99-1 | -9.292 |
| 20 | Hydrangenol | 480-47-7 | -9.214 |
| 21 | Theaflavin-3-Gallate | 30462-34-1 | -9.214 |
| 22 | Trifolirhizin | 6807-83-6 | -9.189 |
| 23 | Okanin | 484-76-4 | -9.132 |
| 24 | Epimedin B | 110623-73-9 | -9.13 |
| 25 | Methylophiopogonone A | 74805-90-6 | -9.117 |
| 26 | 3,4-Dihydroxyflavone | 4143-64-0 | -9.112 |
| 27 | 6-Hydroxyflavanone | 4250-77-5 | -9.091 |
| 28 | Licochalcone A | 58749-22-7 | -9.071 |
| 29 | S-Dihydrodaidzein | 879559-75-8 | -9.053 |
| 30 | Kaempferitrin | 482-38-2 | -9.027 |
| 31 | Kaempferol 3-O-gentiobioside | 22149-35-5 | -9.005 |
| 32 | 4-Hydroxyflavanone | 6515-37-3 | -9 |
| 33 | Puerarin 6''-O-Xyloside | 114240-18-5 | -8.996 |
| 34 | Narirutin | 14259-46-2 | -8.983 |
| 35 | Daidzein | 486-66-8 | -8.977 |
| 36 | Flavanomarein | 577-38-8 | -8.97 |
| 37 | Glabrone | 60008-02-8 | -8.955 |
| 38 | Keracyanin chloride | 18719-76-1 | -8.953 |
| 39 | 6-Hydroxyflavone | 6665-83-4 | -8.945 |
| 40 | Didymin | 14259-47-3 | -8.94 |
| 41 | kuwanon G | 75629-19-5 | -8.934 |
| 42 | Silychristin | 33889-69-9 | -8.928 |
| 43 | 6,2'-Dihydroxyflavone | 92439-20-8 | -8.928 |
| 44 | Ternatumoside II | 1473419-87-2 | -8.923 |
| 45 | Corylin | 53947-92-5 | -8.884 |
| 46 | Astilbin | 29838-67-3 | -8.873 |
| 47 | Dihydrodaidzein | 17238-05-0 | -8.864 |
| 48 | Isobavachin | 31524-62-6 | -8.864 |
| 49 | Maackiain | 19908-48-6 | -8.851 |
| 50 | Rutin | 153-18-4 | -8.847 |
| 51 | Quercetin 3-gentiobioside | 7431-83-6 | -8.846 |
| 52 | Medicarpin | 32383-76-9 | -8.832 |
| 53 | 5-Hydroxyflavone | 491-78-1 | -8.826 |
| 54 | 6''-O-xylosyl-glycitin | 231288-18-9 | -8.812 |
| 55 | Isookanin | 1036-49-3 | -8.807 |
| 56 | Desmethylglycitein | 17817-31-1 | -8.789 |
| 57 | 3'-Hydroxypuerarin | 117060-54-5 | -8.786 |
| 58 | 6-Hydroxyluteolin 7-glucoside | 54300-65-1 | -8.781 |
| 59 | 4-Hydroxychalcone | 20426-12-4 | -8.774 |
| 60 | Afzelin | 482-39-3 | -8.752 |
| 61 | Flavanone | 487-26-3 | -8.743 |
| 62 | Calycosin-7-O-beta-D-glucoside | 20633-67-4 | -8.74 |
| 63 | 7,8,3',4'-tetrahydroxyflavanone | 489-73-6 | -8.731 |
| 64 | 7-Hydroxyisoflavone | 13057-72-2 | -8.726 |
| 65 | Isosilybin | 72581-71-6 | -8.717 |
| 66 | 6-Methylflavone | 29976-75-8 | -8.717 |
| 67 | Liquiritigenin | 578-86-9 | -8.711 |
| 68 | Mirificin | 103654-50-8 | -8.711 |
| 69 | (-)-Butin | 492-14-8 | -8.706 |
| 70 | Genistein | 446-72-0 | -8.705 |
| 71 | Sec-O-Glucosylhamaudol | 80681-44-3 | -8.704 |
| 72 | 2',4,4',6'-Tetrahydroxychalcone | 73692-50-9 | -8.696 |
| 73 | Echinatin | 34221-41-5 | -8.693 |
| 74 | Kaempferol 3-neohesperidoside | 32602-81-6 | -8.678 |
| 75 | Naringenin chalcone | 25515-46-2 | -8.669 |
| 76 | Flavone | 525-82-6 | -8.652 |
| 77 | Isovitexin | 38953-85-4 | -8.65 |
| 78 | Butein | 487-52-5 | -8.644 |
| 79 | Liquiritin apioside | 74639-14-8 | -8.64 |
| 80 | Glabridin | 59870-68-7 | -8.638 |
| 81 | Silymarin | 65666-07-1 | -8.637 |
| 82 | Ginkgetin | 481-46-9 | -8.618 |
| 83 | 7,4'-Dihydroxyflavone | 2196-14-7 | -8.608 |
| 84 | Neohesperidin Dihydrochalcone | 20702-77-6 | -8.607 |
| 85 | (-)-Catechin gallate | 130405-40-2 | -8.603 |
| 86 | 2-Hydroxyflavanone | 17348-76-4 | -8.591 |
| 87 | Scutellarein | 529-53-3 | -8.578 |
| 88 | 2-Hydroxychalcone | 644-78-0 | -8.578 |
| 89 | Xanthone | 90-47-1 | -8.577 |
| 90 | Isoliquiritigenin | 961-29-5 | -8.569 |
| 91 | Icariin | 489-32-7 | -8.565 |
| 92 | Baicalin | 21967-41-9 | -8.564 |
| 93 | (-)-Epicatechin | 490-46-0 | -8.563 |
| 94 | 3',4',7-Trihydroxyflavone | 2150-11-0 | -8.549 |
| 95 | 3,4-Dimethoxychalcone | 5416-71-7 | -8.547 |
| 96 | 7,8-Dihydroxyflavone | 38183-03-8 | -8.535 |
| 97 | Sophoricoside | 152-95-4 | -8.52 |
| 98 | 7-Hydroxyflavanone | 6515-36-2 | -8.517 |
| 99 | Licochalcone B | 58749-23-8 | -8.49 |
| 100 | Schaftoside | 51938-32-0 | -8.457 |
| 101 | Isoorientin | 4261-42-1 | -8.45 |
| 102 | Morusin | 62596-29-6 | -8.444 |
| 103 | Glycitin | 40246-10-4 | -8.442 |
| 104 | Glabrol | 59870-65-4 | -8.439 |
| 105 | Apigenin | 520-36-5 | -8.429 |
| 106 | Isomucronulatol 7-O-glucoside | 136087-29-1 | -8.421 |
| 107 | Quercetin | 117-39-5 | -8.421 |
| 108 | Isosakuranin | 491-69-0 | -8.41 |
| 109 | Eriocitrin | 13463-28-0 | -8.408 |
| 110 | Methylophiopogonanone B | 74805-91-7 | -8.397 |
| 111 | 7-Hydroxyflavone | 6665-86-7 | -8.393 |
| 112 | Hyperoside | 482-36-0 | -8.39 |
| 113 | Iristectorigenin A | 39012-01-6 | -8.38 |
| 114 | Baicalein | 491-67-8 | -8.38 |
| 115 | Medroxyprogesterone Acetate | 71-58-9 | -8.379 |
| 116 | Methyl-Hesperidin | 11013-97-1 | -8.359 |
| 117 | Oroxin B | 114482-86-9 | -8.353 |
| 118 | trans-Chalcone | 614-47-1 | -8.343 |
| 119 | 3,4-Dihydroxyflavone | 4143-64-0 | -8.329 |
| 120 | DiosMetin 7-O-beta-D-Glucuronide | 35110-20-4 | -8.328 |
| 121 | Luteolin-3-O-beta-D-glucuronide | 53527-42-7 | -8.328 |
| 122 | Neoisoastilbin | 54141-72-9 | -8.326 |
| 123 | Pinocembrin | 480-39-7 | -8.324 |
| 124 | 5-methoxyflavone | 42079-78-7 | -8.318 |
| 125 | 2'-O-Galloylhyperin | 53209-27-1 | -8.312 |
| 126 | Quercitrin | 522-12-3 | -8.301 |
| 127 | Nicotiflorin | 17650-84-9 | -8.298 |
| 128 | Silydianin | 29782-68-1 | -8.295 |
| 129 | Liquiritin | 551-15-5 | -8.288 |
| 130 | (-)-Epicatechin gallate | 1257-08-5 | -8.287 |
| 131 | Daidzin | 552-66-9 | -8.285 |
| 132 | Lonicerin | 25694-72-8 | -8.273 |
| 133 | Deguelin | 522-17-8 | -8.269 |
| 134 | 4-Hydroxychalcone | 20426-12-4 | -8.262 |
| 135 | Isoliquiritin apioside | 120926-46-7 | -8.261 |
| 136 | Quercetin-3-O-D-glucosyl-(1-2)-L-rhamnoside | 143016-74-4 | -8.248 |
| 137 | Pinocembrin chalcone | 4197-97-1 | -8.247 |
| 138 | Sciadopitysin | 521-34-6 | -8.247 |
| 139 | 2'-Methoxyflavone | 19725-47-4 | -8.247 |
| 140 | Naringenin | 480-41-1 | -8.236 |
| 141 | 3,6-Dihydroxyflavone | 108238-41-1 | -8.233 |
| 142 | Chalcone | 94-41-7 | -8.229 |
| 143 | Ligustroflavone | 260413-62-5 | -8.227 |
| 144 | 2-Hydroxychalcone | 644-78-0 | -8.224 |
| 145 | Eriodictyol | 552-58-9 | -8.207 |
| 146 | alpha-Naphthoflavone | 604-59-1 | -8.202 |
| 147 | Naringin | 10236-47-2 | -8.194 |
| 148 | Poriol | 14348-16-4 | -8.192 |
| 149 | 6-Methoxyflavone | 26964-24-9 | -8.189 |
| 150 | Icariside I | 56725-99-6 | -8.183 |
| 151 | Chrysin | 480-40-0 | -8.175 |
| 152 | Tectorigenin 7-O-xylosylglucoside | 231288-19-0 | -8.174 |
| 153 | Isoflavone | 574-12-9 | -8.169 |
| 154 | Neohesperidin | 13241-33-3 | -8.161 |
| 155 | Flavonol | 577-85-5 | -8.16 |
| 156 | Luteolin 7-O-glucuronide | 29741-10-4 | -8.144 |
| 157 | Tectoridin | 611-40-5 | -8.143 |
| 158 | Chrysin-7-O-glucuronide | 35775-49-6 | -8.14 |
| 159 | Calycosin | 20575-57-9 | -8.132 |
| 160 | 1-(2,6-dimethoxyphenyl)-3-(4-hydroxyphenyl) propan-1-one | 221696-69-1 | -8.127 |
| 161 | Oroxylin A-7-O-glucuronide | 36948-76-2 | -8.115 |
| 162 | Corylifol A | 775351-88-7 | -8.108 |
| 163 | 17α-Hydroxyprogesterone | 68-96-2 | -8.108 |
| 164 | Vincetoxicoside B | 22007-72-3 | -8.103 |
| 165 | (+/-)-Naringenin | 67604-48-2 | -8.1 |
| 166 | Ombuoside | 20188-85-6 | -8.096 |
| 167 | Ononin | 486-62-4 | -8.086 |
| 168 | Pinocembrin 7-O-beta-D-glucoside | 75829-43-5 | -8.083 |
| 169 | Swertisin | 1859726 | -8.078 |
| 170 | Procyanidin C1 | 37064-30-5 | -8.076 |
| 171 | Plantagoside | 78708-33-5 | -8.073 |
| 172 | Norwogonin | 929064 | -8.072 |
| 173 | Neoliquiritin | 5088-75-5 | -8.066 |
| 174 | 3-Methoxyflavone | 1952259 | -8.064 |
| 175 | Neoeriocitrin | 13241-32-2 | -8.054 |
| 176 | Isorhamnetin-3-O-glucoside | 5041-82-7 | -8.049 |
| 177 | Fisetin | 528-48-3 | -8.046 |
| 178 | Loureirin C | 116384-24-8 | -8.045 |
| 179 | 3'-Methoxypuerarin | 117047-07-1 | -8.044 |
| 180 | Licochalcone D | 144506-15-0 | -8.044 |
| 181 | beta-Anhydroicaritin | 38226-86-7 | -8.037 |
| 182 | Rhoifolin | 17306-46-6 | -8.036 |
| 183 | Quercetagetin | 90-18-6 | -8.034 |
| 184 | Bavachalcone | 28448-85-3 | -8.029 |
| 185 | Tiliroside | 20316-62-5 | -8.027 |
| 186 | Rotenone | 83-79-4 | -8.026 |
| 187 | Glycyrrhisoflavone | 116709-70-7 | -8.022 |
| 188 | Scutellarin methyl ester | 119262-68-9 | -8.011 |
| 189 | Luteolin | 491-70-3 | -8.009 |
| 190 | Wogonoside | 51059-44-0 | -8.009 |
| 191 | Isoliquiritin | 5041-81-6 | -8.007 |
| 192 | Spinosin | 72063-39-9 | -8.005 |
| 193 | Isoastilbin | 54081-48-0 | -8.005 |
| 194 | (-)-Epigallocatechin Gallate | 989-51-5 | -8.005 |
| 195 | Maohuoside A | 128988-55-6 | -7.999 |
| 196 | Scutellarin | 27740-01-8 | -7.998 |
| 197 | Miquelianin | 22688-79-5 | -7.998 |
| 198 | Trilobatin | 4192-90-9 | -7.989 |
| 199 | Hesperetin 7-O-glucoside | 31712-49-9 | -7.969 |
| 200 | Puerarin | 3681-99-0 | -7.962 |
| 201 | Pratensein | 2284-31-3 | -7.952 |
| 202 | 3'-Methoxyapiin | 33579-63-4 | -7.949 |
| 203 | Dichotomitin | 88509-91-5 | -7.935 |
| 204 | Astragalin | 480-10-4 | -7.924 |
| 205 | Oroxin A | 57396-78-8 | -7.918 |
| 206 | saponaretin | 29702-25-8 | -7.916 |
| 207 | Phellamurin | 52589-11-4 | -7.904 |
| 208 | Linarin | 480-36-4 | -7.886 |
| 209 | Robinin | 301-19-9 | -7.883 |
| 210 | Chamaejasmenin B | 89595-71-1 | -7.875 |
| 211 | Prunin | 529-55-5 | -7.871 |
| 212 | Cynaroside | 1268798 | -7.87 |
| 213 | Methylophiopogonanone A | 74805-92-8 | -7.862 |
| 214 | Leucoside | 27661-51-4 | -7.856 |
| 215 | Wushanicaritin | 521-45-9 | -7.853 |
| 216 | Bavachin | 19879-32-4 | -7.85 |
| 217 | Kaempferol | 520-18-3 | -7.841 |
| 218 | Vicenin 3 | 59914-91-9 | -7.838 |
| 219 | Kaempferol-3-O-glucosyl(1-2)rhamnoside | 142451-65-8 | -7.835 |
| 220 | Baicalin methyl ester | 82475-03-4 | -7.828 |
| 221 | Diosmetin 7-O-beta-D-glucopyranoside | 20126-59-4 | -7.825 |
| 222 | (-)-Epigallocatechin Gallate | 989-51-5 | -7.82 |
| 223 | Dihydrorotenone | 6659-45-6 | -7.818 |
| 224 | Epimedoside A | 39012-04-9 | -7.813 |
| 225 | 4'-METHOXYFLAVONE | 4143-74-2 | -7.813 |
| 226 | Isokurarinone | 52483-02-0 | -7.813 |
| 227 | Wogonin | 632-85-9 | -7.81 |
| 228 | Hemiphloin | 71963-94-5 | -7.808 |
| 229 | Diosmin | 520-27-4 | -7.797 |
| 230 | Kuromanin chloride | 7084-24-4 | -7.79 |
| 231 | Biochanin A | 491-80-5 | -7.789 |
| 232 | Quercimeritrin | 491-50-9 | -7.785 |
| 233 | 4'-Methoxypuerarin | 92117-94-7 | -7.783 |
| 234 | Bavachinin | 19879-30-2 | -7.768 |
| 235 | Apiin | 26544-34-3 | -7.762 |
| 236 | Apigenin-7-glucuronide | 29741-09-1 | -7.758 |
| 237 | Swertiajaponin | 6980-25-2 | -7.757 |
| 238 | Sinensin | 28189-90-4 | -7.752 |
| 239 | Linaroside | 53452-12-3 | -7.752 |
| 240 | Icaritin | 118525-40-9 | -7.742 |
| 241 | Isoengeletin | 30987-58-7 | -7.74 |
| 242 | 3',4'-Dimethoxyflavone | 4143-62-8 | -7.73 |
| 243 | Juglanin | 5041-67-8 | -7.728 |
| 244 | Loureirin A | 119425-89-7 | -7.727 |
| 245 | Millepachine | 1393922-01-4 | -7.724 |
| 246 | Kaempferol 3,4',7-triacetate | 143724-69-0 | -7.721 |
| 247 | Neochamaejasmine B | 90411-12-4 | -7.712 |
| 248 | Oroxylin A-7-O-glucuronide | 36948-76-2 | -7.71 |
| 249 | Neoisoliquiritin | 59122-93-9 | -7.709 |
| 250 | Isobavachromene | 52801-22-6 | -7.708 |
| 251 | (-)-Gallocatechin gallate | 4233-96-9 | -7.703 |
| 252 | Robinetin | 490-31-3 | -7.703 |
| 253 | Licochalcone C | 144506-14-9 | -7.686 |
| 254 | Avicularin | 572-30-5 | -7.681 |
| 255 | Catechin | 154-23-4 | -7.677 |
| 256 | Dracorhodin perchlorate | 125536-25-6 | -7.676 |
| 257 | Reynoutrin | 549-32-6 | -7.667 |
| 258 | (E)-Cardamonin | 19309-14-9 | -7.658 |
| 259 | Neosmitilbin | 54081-47-9 | -7.654 |
| 260 | 2''-O-Rhamnosylicariside II | 135293-13-9 | -7.651 |
| 261 | Kakkalide | 58274-56-9 | -7.65 |
| 262 | Irisolidone | 2345-17-7 | -7.65 |
| 263 | Rhodionin | 85571-15-9 | -7.649 |
| 264 | Hesperetin | 520-33-2 | -7.641 |
| 265 | 3'-Methoxyflavonol | 76666-32-5 | -7.633 |
| 266 | (+)-catechin | 7295-85-4 | -7.629 |
| 267 | Acacetin | 480-44-4 | -7.624 |
| 268 | Orientin | 28608-75-5 | -7.614 |
| 269 | Apigenin 7-glucoside | 578-74-5 | -7.606 |
| 270 | Isoginkgetin | 548-19-6 | -7.602 |
| 271 | Isosakuranetin | 480-43-3 | -7.6 |
| 272 | Tricetin | 520-31-0 | -7.592 |
| 273 | Kaempferol-3-O-glucorhamnoside | 40437-72-7 | -7.573 |
| 274 | coreopsin | 499-29-6 | -7.559 |
| 275 | 7-Methoxyisoflavone | 1621-56-3 | -7.558 |
| 276 | Taxifolin | 480-18-2 | -7.557 |
| 277 | Rutin hydrate | 207671-50-9 | -7.556 |
| 278 | (+)-Catechin Hydrate | 225937-10-0 | -7.554 |
| 279 | Guaijaverin | 22255-13-6 | -7.538 |
| 280 | 4-methyl-6-phenyl-2H-pyranone | 4467-30-5 | -7.537 |
| 281 | Quercetin Dihydrate | 6151-25-3 | -7.533 |
| 282 | Aureusidin | 38216-54-5 | -7.532 |
| 283 | Homoplantaginin | 17680-84-1 | -7.524 |
| 284 | (+)-Taxifolin | 24198-97-8 | -7.51 |
| 285 | Irisflorentin | 41743-73-1 | -7.509 |
| 286 | Helichrysetin | 62014-87-3 | -7.508 |
| 287 | Neobavaisoflavone | 41060-15-5 | -7.504 |
| 288 | Tectorigenin | 548-77-6 | -7.503 |
| 289 | Engeletin | 572-31-6 | -7.501 |
| 290 | Hispidulin | 1447-88-7 | -7.5 |
| 291 | Xanthohumol | 6754-58-1 | -7.494 |
| 292 | Aloesin | 30861-27-9 | -7.491 |
| 293 | Iridin | 491-74-7 | -7.49 |
| 294 | Kaempferol-7-o-beta-D-glucopyranoside | 16290-07-6 | -7.483 |
| 295 | Breviscapin | 116122-36-2 | -7.483 |
| 296 | Pratol | 487-24-1 | -7.471 |
| 297 | Isoquercetin | 482-35-9 | -7.465 |
| 298 | Rhodiosin | 86831-54-1 | -7.463 |
| 299 | Nobiletin | 478-01-3 | -7.456 |
| 300 | Prunetin | 552-59-0 | -7.455 |
| 301 | Galangin | 548-83-4 | -7.443 |
| 302 | kaempferide | 491-54-3 | -7.438 |
| 303 | Glycitein | 40957-83-3 | -7.437 |
| 304 | Prenyletin | 15870-91-4 | -7.428 |
| 305 | Farrerol | 24211-30-1 | -7.427 |
| 306 | Marein | 535-96-6 | -7.423 |
| 307 | Tricin | 520-32-1 | -7.417 |
| 308 | Irigenin | 548-76-5 | -7.414 |
| 309 | 4',7-Dimethoxyisoflavone | 1157-39-7 | -7.409 |
| 310 | (-)-catechin | 18829-70-4 | -7.404 |
| 311 | Isorhamnetin | 480-19-3 | -7.401 |
| 312 | 7,3',4'-Tri-O-methylluteolin | 29080-58-8 | -7.395 |
| 313 | (2S)-Isoxanthohumol | 70872-29-6 | -7.394 |
| 314 | Myricetin | 529-44-2 | -7.383 |
| 315 | Baohuoside I | 113558-15-9 | -7.38 |
| 316 | Troxerutin | 7085-55-4 | -7.376 |
| 317 | Myricitrin | 17912-87-7 | -7.36 |
| 318 | Sanggenon C | 80651-76-9 | -7.35 |
| 319 | Formononetin | 485-72-3 | -7.347 |
| 320 | Morin | 480-16-0 | -7.346 |
| 321 | Theaflavin-3-gallate | 30462-34-1 | -7.338 |
| 322 | Casticin | 479-91-4 | -7.33 |
| 323 | Dihydrokaempferol | 480-20-6 | -7.328 |
| 324 | Tilianin | 4291-60-5 | -7.328 |
| 325 | 4',7-Di-O-methylnaringenin | 29424-96-2 | -7.321 |
| 326 | Chrysoeriol | 491-71-4 | -7.319 |
| 327 | Negletein | 29550-13-8 | -7.318 |
| 328 | Phlorizin dihydrate | 7061-54-3 | -7.302 |
| 329 | Colutehydroquinone | 181311-16-0 | -7.302 |
| 330 | Hydroxysafflor yellow A | 78281-02-4 | -7.298 |
| 331 | Quercetagitrin | 548-75-4 | -7.295 |
| 332 | Pectolinarin | 28978-02-1 | -7.294 |
| 333 | Pinostrobin | 480-37-5 | -7.287 |
| 334 | Dihydromyricetin | 27200-12-0 | -7.28 |
| 335 | Lupiwighteone | 104691-86-3 | -7.278 |
| 336 | Alpinetin | 36052-37-6 | -7.264 |
| 337 | Persicogenin | 28590-40-1 | -7.262 |
| 338 | Salvigenin | 19103-54-9 | -7.258 |
| 339 | 4-Hydroxycoumarin | 1076-38-6 | -7.256 |
| 340 | Genkwanin | 437-64-9 | -7.248 |
| 341 | Brazilin | 474-07-7 | -7.242 |
| 342 | 8-Prenylnaringenin | 53846-50-7 | -7.227 |
| 343 | Visnagin | 82-57-5 | -7.226 |
| 344 | Oroxylin A | 480-11-5 | -7.225 |
| 345 | Homobutein | 34000-39-0 | -7.223 |
| 346 | Nepetin | 520-11-6 | -7.212 |
| 347 | Sakuranetin | 2957-21-3 | -7.207 |
| 348 | Cyanidin Chloride | 528-58-5 | -7.206 |
| 349 | Pectolinarigenin | 520-12-7 | -7.19 |
| 350 | herbacetin-3,8-diglucopyranoside | 99224-12-1 | -7.189 |
| 351 | Baimaside | 18609-17-1 | -7.187 |
| 352 | Diosmetin | 520-34-3 | -7.167 |
| 353 | Kurarinone | 34981-26-5 | -7.166 |
| 354 | 3-O-Methylgalangin | 6665-74-3 | -7.164 |
| 355 | Hamaudol | 735-46-6 | -7.156 |
| 356 | Padmatin | 80453-44-7 | -7.153 |
| 357 | Skullcapflavone II | 55084-08-7 | -7.148 |
| 358 | 1-[2,4-Dihydroxy-6-methoxy-3-(3-methyl-2-buten-1-yl) phenyl]-3-(4-hydroxyphenyl)-2-propen-1-one | 569-83-5 | -7.147 |
| 359 | 6-Demethoxytangeretin | 6601-66-7 | -7.146 |
| 360 | Theaflavin | 1011850 | -7.143 |
| 361 | 5,7,4'-Trimethoxyflavone | 5631-70-9 | -7.143 |
| 362 | Azaleatin | 529-51-1 | -7.14 |
| 363 | Vitexin | 3681-93-4 | -7.132 |
| 364 | Jaceosidin | 18085-97-7 | -7.127 |
| 365 | Prudomestin | 3443-28-5 | -7.125 |
| 366 | 4-Methoxychalcone | 959-33-1 | -7.115 |
| 367 | Chrysosplenol D | 14965-20-9 | -7.113 |
| 368 | (-)-Gallocatechin | 3371-27-5 | -7.105 |
| 369 | Angelicain | 49624-66-0 | -7.099 |
| 370 | 6-Methoxynaringenin | 94942-49-1 | -7.079 |
| 371 | Nevadensin | 10176-66-6 | -7.073 |
| 372 | Blumeatin | 118024-26-3 | -7.072 |
| 373 | 5,7-Dimethoxyluteolin | 90363-40-9 | -7.071 |
| 374 | Eupatorin | 855-96-9 | -7.071 |
| 375 | Chrysosplenetin | 603-56-5 | -7.069 |
| 376 | Isosinensetin | 17290-70-9 | -7.069 |
| 377 | Neodiosmin | 38665-01-9 | -7.068 |
| 378 | Isobavachalcone | 20784-50-3 | -7.064 |
| 379 | Isorhamnetin-3-O-neohespeidoside | 55033-90-4 | -7.063 |
| 380 | Amentoflavone | 1617-53-4 | -7.061 |
| 381 | Tamarixetin | 603-61-2 | -7.058 |
| 382 | Ginkgolide C | 15291-76-6 | -7.058 |
| 383 | Mosloflavone | 740-33-0 | -7.055 |
| 384 | 5-Desmethylsinensetin | 21763-80-4 | -7.043 |
| 385 | Sinensetin | 2306-27-6 | -7.039 |
| 386 | Naringenin trimethyl ether | 38302-15-7 | -7.036 |
| 387 | Mulberrin | 62949-79-5 | -7.032 |
| 388 | 5-Methyl-7-methoxyisoflavone | 82517-12-2 | -7.023 |
| 389 | 5,7-Dihydroxychromone | 31721-94-5 | -7.019 |
| 390 | 7-Methoxyflavone | 22395-22-8 | -7.015 |
| 391 | Flavokawain A | 3420-72-2 | -7.01 |
| 392 | Flavokawain B | 1775-97-9 | -7.009 |
| 393 | 4'-O-Methylbavachalcone | 20784-60-5 | -7.005 |
| 394 | Lysionotin | 152743-19-6 | -7.003 |
| 395 | 3,5,6,7,8,3',4'-Heptamethoxyflavone | 1178-24-1 | -6.995 |
| 396 | Ayanin | 572-32-7 | -6.974 |
| 397 | Eupatilin | 22368-21-4 | -6.973 |
| 398 | 3',5-Di-O-methyl-quercetin | 40554-94-7 | -6.963 |
| 399 | Typhaneoside | 104472-68-6 | -6.956 |
| 400 | Artemitin | 479-90-3 | -6.948 |
| 401 | Sophoraflavanone G | 97938-30-2 | -6.946 |
| 402 | 4',6,7-Trimethoxyisoflavone | 798-61-8 | -6.932 |
| 403 | Rhamnetin | 90-19-7 | -6.931 |
| 404 | Tetramethylkaempferol | 16692-52-7 | -6.93 |
| 405 | Flavokawain A | 3420-72-2 | -6.929 |
| 406 | Procyanidin B3 | 23567-23-9 | -6.926 |
| 407 | Tectochrysin | 520-28-5 | -6.925 |
| 408 | Epmedin C | 110642-44-9 | -6.917 |
| 409 | Hibifolin | 55366-56-8 | -6.906 |
| 410 | Herbacetin | 527-95-7 | -6.901 |
| 411 | Karanjin | 521-88-0 | -6.884 |
| 412 | Moslosooflavone | 3570-62-5 | -6.882 |
| 413 | Isoschaftoside | 52012-29-0 | -6.878 |
| 414 | 5,7,3'-Trihydroxy-6,4',5'-trimethoxyflavone | 78417-26-2 | -6.873 |
| 415 | 5,6,7-TRIMETHOXYFLAVONE | 973-67-1 | -6.859 |
| 416 | 5-O-Demethylnobiletin | 2174-59-6 | -6.856 |
| 417 | MRS928 | 26964-29-4 | -6.85 |
| 418 | (-)-Gallocatechin | 3371-27-5 | -6.842 |
| 419 | Kaempferol 3-O-sophoroside | 19895-95-5 | -6.832 |
| 420 | Flavokawain C | 37308-75-1 | -6.831 |
| 421 | 5,7,3',4'-Tetramethoxyflavone | 855-97-0 | -6.821 |
| 422 | 3',4',5',5,7-Pentamethoxyflavone | 53350-26-8 | -6.794 |
| 423 | 2',4,4',6'-Tetramethoxychalcone | 94103-36-3 | -6.788 |
| 424 | Aloeresin D | 105317-67-7 | -6.78 |
| 425 | Noricaritin | 5240-95-9 | -6.773 |
| 426 | Isoneochamaejasmin A | 871319-96-9 | -6.745 |
| 427 | Kaempferol 3,4',7-triacetate | 143724-69-0 | -6.744 |
| 428 | 3',4',7-Trimethoxyquercetin | 6068-80-0 | -6.735 |
| 429 | Kaempferol 3,4',7-triacetate | 143724-69-0 | -6.726 |
| 430 | Retusin | 1245-15-4 | -6.713 |
| 431 | Cimifugin | 37921-38-3 | -6.696 |
| 432 | Khellin | 82-02-0 | -6.695 |
| 433 | EGCG Octaacetate | 148707-39-5 | -6.671 |
| 434 | Scutellarein tetramethyl ether | 1168-42-9 | -6.663 |
| 435 | 5,7-DIMETHOXYFLAVONE | 21392-57-4 | -6.661 |
| 436 | Hydroxygenkwanin | 20243-59-8 | -6.637 |
| 437 | Kumatakenin | 3301-49-3 | -6.635 |
| 438 | Vaccarin | 53452-16-7 | -6.635 |
| 439 | Vicenin 2 | 23666-13-9 | -6.633 |
| 440 | 4,4'-Dimethoxychalcone | 2373-89-9 | -6.607 |
| 441 | Coreoside B | 1580464-83-0 | -6.607 |
| 442 | 5-hydroxy-3,7-dimethoxy-2-phenylchromen | 70786-48-0 | -6.573 |
| 443 | Quercetin-3,5,7,3',4'-pentamethyl ether | 1247-97-8 | -6.573 |
| 444 | Noreugenin | 1013-69-0 | -6.55 |
| 445 | Rhamnocitrin | 569-92-6 | -6.55 |
| 446 | Hinokiflavone | 19202-36-9 | -6.499 |
| 447 | Camelliaside A | 135095-52-2 | -6.46 |
| 448 | Gardenin B | 2798-20-1 | -6.358 |
| 449 | Procyanidin A1 | 103883-03-0 | -6.336 |
| 450 | Procyanidin A2 | 41743-41-3 | -6.273 |
| 451 | 6'''-Feruloylspinosin | 77690-92-7 | -6.243 |
| 452 | Loureirin B | 119425-90-0 | -6.234 |
| 453 | Gossypin | 652-78-8 | -6.201 |
| 454 | 7,2'-Dihydroxy-3',4'-dimethoxyisoflavan 7-O-beta-D-glucoside | 94367-43-8 | -6.116 |
| 455 | Procyanidin B1 | 20315-25-7 | -6.114 |
| 456 | Phlorizin | 60-81-1 | -6.094 |
| 457 | Sinocrassoside C1 | 909803-26-5 | -6.016 |
| 458 | Tangeretin | 481-53-8 | -5.997 |
| 459 | 2'-O-beta-L-galactopyranosylorientin | 861691-37-4 | -5.936 |
| 460 | Complanatuside | 116183-66-5 | -5.934 |
| 461 | Narcissin | 604-80-8 | -5.922 |
| 462 | Taxifolin 7-O-rhamnoside | 137592-12-2 | -5.889 |
| 463 | Proanthocyanidins | 20347-71-1 | -5.852 |
| 464 | Tribuloside | 22153-44-2 | -5.636 |
| 465 | Gastrodenol | 57644-54-9 | -5.626 |
| 466 | 1,3,5-Trimethoxybenzene | 621-23-8 | -5.394 |
| 467 | Maltol | 118-71-8 | -5.343 |
| 468 | Complanatoside B | 142473-99-2 | -5.122 |
| 469 | Pinocembrin 7-O-(3''-galloyl-4'',6''-(S)-hexahydroxydiphenoyl)-beta-D-glucose | 205370-59-8 | -4.978 |
| 470 | Thonningianin A | 271579-11-4 | -4.773 |
| 471 | Apigenin-7-O-(2G-rhamnosyl) gentiobioside | 174284-20-9 | -4.584 |
| 472 | Theaflavine-3,3'-digallate | 33377-72-9 | -4.025 |
| 473 | (2R)-2,3-Dihydro-5,7-dihydroxy-2-(4-hydroxyphenyl)-6-methoxy-4H-1-benzopyran-4-one | 1212075-61-0 | -3.76 |
| 474 | Theaflavin 3,3'-digallate | 30462-35-2 | -2.855 |
| 475 | Epimedin A | 110623-72-8 | 2.409 |
